# Supplementary material for: Computing conditional entropies for quantum correlations
Source: Nat Commun. 2021 Jan 25;12:575. doi: 10.1038/s41467-020-20018-1 (PMC7835250; doi:10.1038/s41467-020-20018-1)
Supplement: Supplementary file 1 — Supplementary Information [file 41467_2020_20018_MOESM1_ESM.pdf]

Computing conditional entropies for quantum correlations
Supplementary Information

Peter Brown, Hamza Fawzi and Omar Fawzi

October 27, 2020

Preliminaries

In order to keep this document as self-contained as possible we repeat the preliminaries from the main text as well as the definitions for the iterated mean divergences and corresponding conditional entropies. We define $\mathbb{N}$ to be the set of strictly positive integers. Let $\mathcal{H}$ be a Hilbert space; we denote the set of linear operators on $\mathcal{H}$ by $\mathscr{L}(\mathcal{H})$, the set of Hermitian operators on $\mathcal{H}$ by $\mathscr{H}(\mathcal{H})$, the set of positive semidefinite operators on $\mathcal{H}$ by $\mathscr{P}(\mathcal{H})$ and the set of positive semidefinite operators with unit trace on $\mathcal{H}$ by $\mathscr{D}(\mathcal{H})$. All Hilbert spaces in this work are finite dimensional unless otherwise stated. Given a linear map $\mathcal{E} : \mathscr{L}(\mathcal{H}_1) \to \mathscr{L}(\mathcal{H}_2)$, we say $\mathcal{E}$ is CPTP if it is completely positive and trace preserving. Given two Hilbert spaces $\mathcal{H}$ and $\mathcal{K}$ we write $\mathcal{H}\mathcal{K}$ as shorthand for $\mathcal{H} \otimes \mathcal{K}$. Given two operators $A, B \in \mathscr{L}(\mathcal{H})$ we write $A \leq B$ if $B - A \in \mathscr{P}(\mathcal{H})$. The support of an operator $A \in \mathscr{L}(\mathcal{H})$, denoted $\mathrm{supp}(A)$, is the orthogonal complement of its kernel, $\ker(A) = \{x \in \mathcal{H} : Ax = 0\}$. For $A, B \in \mathscr{L}(\mathcal{H})$, we write $A \ll B$ if $\mathrm{supp}(A) \subseteq \mathrm{supp}(B)$. For $A \in \mathscr{L}(\mathcal{H})$, A^* denotes its adjoint and if A is nonsingular then A^{-1} denotes its inverse. If A is singular then A^{-1} denotes the Moore-Penrose pseudo-inverse of A. We use the symbol I to denote the identity operator. A collection of operators $\{M_1, \dots, M_n\}$ forms an n-outcome POVM on $\mathcal{H}$ if $\sum_{i=1}^n M_i = I$ and $M_i \in \mathscr{P}(\mathcal{H})$ for all $i = 1, \dots, n$. Throughout this work we shall be interested in classical systems that arise from measurements on some quantum system. To distinguish the classical and quantum systems in this process we shall often write a single uppercase Roman character to denote the classical system, e.g. A, and the denote the corresponding quantum system from which it is obtained by Q_A.

The geometric mean of two positive definite matrices A and B is defined as

$$A\#B = A^{1/2}(A^{-1/2}BA^{-1/2})^{1/2}A^{1/2}. \tag{1}$$

This definition can be extended to positive semidefinite matrices A, B as $\lim_{\epsilon\to 0} A_\epsilon \# B_\epsilon$ where $X_\epsilon = X + \epsilon I$. The geometric mean has the property that if $C \leq D$ then $A\#C \leq A\#D$ [1, Corollary 3.2.3].

Let $\alpha \in (0,1) \cup (1,\infty)$, $\rho \in \mathscr{D}(\mathcal{H})$ and $\sigma \in \mathscr{P}(\mathcal{H})$ with $\rho \ll \sigma$. The Petz-Rényi divergence [2] of order α is defined as

$$\overline{D}_\alpha(\rho\|\sigma) := \frac{1}{\alpha - 1} \log \mathrm{Tr}\left[\rho^\alpha \sigma^{1-\alpha}\right]. \tag{2}$$

The sandwiched Rényi divergence [3, 4] of order α is defined as

$$\widetilde{D}_\alpha(\rho\|\sigma) := \frac{1}{\alpha - 1} \log \mathrm{Tr}\left[\left(\sigma^{\frac{1-\alpha}{2\alpha}} \rho \sigma^{\frac{1-\alpha}{2\alpha}}\right)^\alpha\right]. \tag{3}$$

In the limit $\alpha \to 1$ both the Petz-Rényi divergence and the sandwiched Rényi divergence converge to the Umegaki relative entropy [5]

$$D(\rho\|\sigma) := \mathrm{Tr}\left[\rho(\log\rho - \log\sigma)\right]. \tag{4}$$

The geometric Rényi divergence [6] of order α is defined as

$$\widehat{D}_\alpha(\rho\|\sigma) := \frac{1}{\alpha - 1} \log \mathrm{Tr}\left[\rho^{1/2}\left(\rho^{-1/2}\sigma\rho^{-1/2}\right)^{1-\alpha}\rho^{1/2}\right]. \tag{5}$$

In the limit $\alpha \to 1$ the geometric Rényi divergence converges to the Belavkin-Staszewski relative entropy $\mathrm{Tr}\left[\rho \log(\rho^{1/2}\sigma^{-1}\rho^{1/2})\right]$ [7]. The geometric Rényi divergence is the largest Rényi divergence satisfying data-processing. The max divergence is defined as

$$D_{\max}(\rho\|\sigma) := \log \inf\{\lambda > 0 : \rho \leq \lambda\sigma\}. \tag{6}$$

Finally, the measured Rényi divergence is defined as the largest classical divergence obtained from measuring ρ and σ. For $\alpha \in (1, \infty)$ this is formally defined as

$$D_\alpha^{\mathbb{M}}(\rho\|\sigma) := \frac{1}{\alpha - 1} \log \sup_{\{M_i\}_i} \sum_i \mathrm{Tr}\left[M_i\rho\right]^\alpha \mathrm{Tr}\left[M_i\sigma\right]^{1-\alpha}, \tag{7}$$

where the supremum is taken over all POVMs $\{M_i\}$. This divergence also admits the following variational characterization [8]

$$D_\alpha^{\mathbb{M}}(\rho\|\sigma) = \frac{1}{\alpha - 1} \log \sup_{\omega > 0} \alpha \mathrm{Tr}\left[\rho\omega^{1-\frac{1}{\alpha}}\right] + (1-\alpha)\mathrm{Tr}\left[\sigma\omega\right]. \tag{8}$$

Given bipartite state $\rho_{AB} \in \mathscr{D}(AB)$ and a Rényi divergence $\mathbb{D}$ we define a corresponding conditional entropy

$$\mathbb{H}^{\downarrow}(A|B)_\rho := -\mathbb{D}(\rho_{AB}\|I_A \otimes \rho_B) \tag{9}$$

and a corresponding optimized conditional entropy

$$\mathbb{H}^{\uparrow}(A|B)_\rho := \sup_{\sigma_B \in \mathscr{D}(B)} -\mathbb{D}(\rho_{AB}\|I_A \otimes \sigma_B). \tag{10}$$

The min-entropy is defined as

$$H_{\min}(A|B) = \sup_{\sigma_B \in \mathscr{D}(B)} -D_{\max}(\rho_{AB}\|I_A \otimes \sigma_B). \tag{11}$$

For the sequence $\alpha_k := 1 + \frac{1}{2^k - 1}$ for $k \in \mathbb{N}$, the iterated mean divergence of order α_k is defined as

$$D_{(\alpha_k)}(\rho\|\sigma) := \frac{1}{\alpha_k - 1} \log Q_{(\alpha_k)}(\rho\|\sigma), \tag{12}$$

with

$$\begin{aligned} Q_{(\alpha_k)}(\rho\|\sigma) := \max_{V_1,\dots,V_k,Z} \quad & \alpha_k \mathrm{Tr}\left[\rho \frac{(V_1 + V_1^*)}{2}\right] - (\alpha_k - 1)\mathrm{Tr}\left[\sigma Z\right] \\ \text{s.t.} \quad & V_1 + V_1^* \geq 0 \\ & \begin{pmatrix} I & V_1 \\ V_1^* & \frac{(V_2+V_2^*)}{2} \end{pmatrix} \geq 0 \quad \begin{pmatrix} I & V_2 \\ V_2^* & \frac{(V_3+V_3^*)}{2} \end{pmatrix} \geq 0 \quad \cdots \quad \begin{pmatrix} I & V_k \\ V_k^* & Z \end{pmatrix} \geq 0, \end{aligned} \tag{13}$$

where the optimization varies over $V_1, \dots, V_k \in \mathscr{L}(\mathcal{H})$ and $Z \in \mathscr{P}(\mathcal{H})$. Additionally, we may assume that $Z \ll \sigma$ and $V_i \ll \sigma$ for each $i \in \{1, 2, \dots, k\}$. Furthermore, for a bipartite state ρ_{AB} the corresponding optimized conditional entropy can be expressed as

$$H^{\uparrow}_{(\alpha_k)}(A|B)_\rho = \frac{1}{1 - \alpha_k} \log Q^{\uparrow}_{(\alpha_k)}(\rho) \tag{14}$$

where

$$\begin{aligned} Q^{\uparrow}_{(\alpha_k)}(\rho) = \max_{V_1,\dots,V_k} \quad & \left(\mathrm{Tr}\left[\rho \frac{(V_1 + V_1^*)}{2}\right]\right)^{\alpha_k} \\ \text{s.t.} \quad & \mathrm{Tr}_A\left[V_k^* V_k\right] \leq I_B \\ & V_1 + V_1^* \geq 0 \\ & \begin{pmatrix} I & V_1 \\ V_1^* & \frac{(V_2+V_2^*)}{2} \end{pmatrix} \geq 0 \quad \begin{pmatrix} I & V_2 \\ V_2^* & \frac{(V_3+V_3^*)}{2} \end{pmatrix} \geq 0 \quad \cdots \quad \begin{pmatrix} I & V_{k-1} \\ V_{k-1}^* & \frac{(V_k+V_k^*)}{2} \end{pmatrix} \geq 0. \end{aligned} \tag{15}$$

SDP implementation details

The NPA hierarchy

In this subsection we briefly describe how we can use NPA hierarchy to optimize polynomials of bounded operators. For more details we refer the reader to the original paper [9]. Consider a Hilbert space $\mathcal{H}$, a collection of bounded operators on $\mathcal{H}$, $X = (X_1, \ldots, X_n)$ and a state $|\psi\rangle \in \mathcal{H}$. Call the elements in the collection X letters, then a word consists of an arbitrary product of letters and their adjoints. The length of a word is the number of letters in the product. We consider I to be the empty word and define its length to be 0. Let $\mathcal{W}_k$ be the set of all words of length no larger than k. Now consider the matrix Γ whose elements are indexed by words in the set $\mathcal{W}$ and whose (W_1, W_2) element corresponds to

$$\Gamma_{(W_1,W_2)} = \mathrm{Tr}\left[W_1^* W_2 |\psi\rangle\langle\psi|\right]. \tag{16}$$

It was shown in [9] that this matrix is PSD for all $k \in \mathbb{N}$. We refer to such a matrix as a certificate of level k.

Now suppose we are given a conditional probability distribution $p(a, b|x, y)$. We say p has a quantum spatial realization if there exists a Hilbert space $\mathcal{H}$, a state $|\psi\rangle \in \mathcal{H}$ and POVMs $\{M_{a|x}\}$, $\{N_{b|y}\}$ with $[M_{a|x}, N_{b|y}] = 0$ for all (a, b, x, y) such that $p(a, b|x, y) = \mathrm{Tr}\left[M_{a|x}N_{b|y}|\psi\rangle\langle\psi|\right]$. The above construction allows us to derive necessary conditions for a distribution to have a quantum spatial realization. That is, we know if a quantum realization were to exist then for each $k \in \mathbb{N}$ there exists a certificate of level k. Thus, we can look for a positive semidefinite matrix Γ indexed by words on length no larger than k generated from the set $\{I\} \cup \{M_{a|x}\} \cup \{N_{b|y}\}$ which would be compatible with the distribution p. For example, we know constraints such as

$$\Gamma_{(M_{a|x},N_{b|y})} = \Gamma_{(N_{b|y},M_{a|x})} = \Gamma_{(M_{a|x}N_{b|y},I)} = p(a, b|x, y) \tag{17}$$

and

$$\Gamma_{(I,I)} = 1. \tag{18}$$

After imposing all such constraints, finding a completion of the matrix that is positive semidefinite is an SDP and so can be computed efficiently. The authors of [9] also proved a converse statement: if for each $k \in \mathbb{N}$ there exists a certificate of level k then there exists a quantum realization of the probability distribution.

This construction allows us to relax optimization problems of the form

$$\max \mathrm{Tr}\left[m(X)|\psi\rangle\langle\psi|\right] \tag{19}$$

where $m(X)$ is some Hermitian polynomial of bounded operators and the maximization is taken over all Hilbert spaces $\mathcal{H}$, all collections of bounded operators on that Hilbert space and all states $|\psi\rangle \in \mathcal{H}$ to an SDP. We can add tracial constraints, e.g., $\mathrm{Tr}\left[n(X)|\psi\rangle\langle\psi|\right] = c$ for some polynomial $n(X)$, and also operator inequalities to the optimization (19). Given a Hermitian polynomial $q(X) \geq 0$, if we have a quantum realization then the localizing matrix Γ^{loc} indexed by words in $\mathcal{W}_d$ whose entries are given by

$$\Gamma^{\mathrm{loc}}_{(W_1,W_2)} = \mathrm{Tr}\left[W_1^* q(X) W_2 |\psi\rangle\langle\psi|\right] \tag{20}$$

is also PSD. Therefore, for each operator inequality we add to (19) we can relax the optimization by adding an additional localizing matrix.

Further constraints for $H^{\uparrow}_{(2)}(A|E)$

The following proposition, taken from [10], provides a dilation theorem which can be used to simplify some of our device-independent optimizations.

Proposition 1 (Proposition 1. [10]). Let $n \in \mathbb{N}$ and let $\{V_i : 1 \leq i \leq n\}$ be a collection of bounded linear operators on some Hilbert space $\mathcal{H}$ such that $\sum_{i=1}^{n} V_i^* V_i \leq I$. Then there exists a Hilbert space $\mathcal{K}$, such that $\mathcal{H} \subseteq \mathcal{K}$, and a collection of bounded linear operators $\{S_i : 1 \leq i \leq n\}$ on $\mathcal{K}$ satisfying

1. $S_i(\mathcal{H}) \subseteq \mathcal{H}$ for each $i \in \{1, \dots, n\}$.
2. $S_i S_j^* = \delta_{ij} I_{\mathcal{K}}$ for each $i, j \in \{1, \dots, n\}$.
3. $\sum_{i=1}^n S_i^* S_i \leq I_{\mathcal{K}}$.
4. $P_{\mathcal{H}} S_i|_{\mathcal{H}} = V_i$ for each $i \in \{1, \dots, n\}$.

where $P_{\mathcal{H}}$ is the projector onto the subspace $\mathcal{H}$.

The proof of the above proposition, see [10], gives a construction of the operators S_i. Briefly, it states that we find can some (possibly infinite-dimensional) Hilbert space $\mathcal{L}$ such that $\mathcal{K} = \mathcal{H} \oplus \mathcal{L}$ and operators S_i of the form

$$S_i = \begin{pmatrix} V_i & X_i \\ 0 & Y_i \end{pmatrix} \tag{21}$$

for some suitably chosen operators X_i and Y_i.

We now look to apply the this dilation theorem to improve convergence and efficiency of our device-independent optimizations of $H^{\uparrow}_{(\alpha_k)}$. Let us first describe how the above proposition can be used to improve the optimization of $H^{\uparrow}_{(2)}$, afterwards we shall describe the general case. Recall that $\inf H^{\uparrow}_{(2)}(A|E) = -2\log(Q^{\mathrm{DI}}_{(2)})$ where

$$\begin{aligned} Q^{\mathrm{DI}}_{(2)} = &\sup_{\{V_a\}_a, \{M_a\}_a, |\psi\rangle\langle\psi|, Q_A \otimes E} \sum_a \mathrm{Tr}\left[(M_a \otimes \frac{V_a + V_a^*}{2})|\psi\rangle\langle\psi|\right] \\ &\text{s.t.} \qquad \sum_a V_a^* V_a \leq I_E \\ &\qquad\qquad V_a + V_a^* \geq 0 \qquad \text{for each } a \in \mathcal{A} \end{aligned} \tag{22}$$

where the optimization is over all joint Hilbert spaces $Q_A E$, all states $|\psi\rangle \in Q_A E$, all POVMs $\{M_a\}_a$ on Q_A and all collections of linear operators $V_a \in \mathscr{L}(E)$. For the moment we will drop the operator inequalities $V_a + V_a^* \geq 0$ from the optimization and later we shall discuss how to reinsert them. In general this optimization would also be augmented with constraints on the local statistics generated by the POVMs $\{M_a\}$ and likely would also include a second system Q_B with further POVMs. However, we deal with the simpler case here from which the general case follows readily. Furthermore, the SDP relaxations of this problem [9] provide lower bounds on the optimization even when the Hilbert spaces Q_A and E are infinite dimensional.

Now consider a more restricted optimization

$$\begin{aligned} \widehat{Q}^{\mathrm{DI}}_{(2)} = &\sup_{\{S_a\}_a, \{M_a\}_a, |\psi\rangle\langle\psi|, Q_A \otimes \widehat{E}} \sum_a \mathrm{Tr}\left[(M_a \otimes \frac{S_a + S_a^*}{2})|\psi\rangle\langle\psi|\right] \\ &\text{s.t.} \qquad \sum_a S_a^* S_a \leq I_{\widehat{E}} \\ &\qquad\qquad S_a S_b^* = \delta_{ab} I_{\widehat{E}} \qquad \text{for all } a, b \in \mathcal{A}. \end{aligned} \tag{23}$$

By Proposition 1, any feasible point of (22) can be transformed into a feasible point of (23) with the same objective value. Indeed, the proposition states that we can find a larger Hilbert space $\widehat{E} = E \oplus E^{\perp}$, with operators of the form $S_a = \begin{pmatrix} V_a & X_a \\ 0 & Y_a \end{pmatrix}$ satisfying the constraints of (23). Moreover, we can use an isometry $W : E \to \widehat{E}$ to embed the state $|\psi\rangle \in Q_A \otimes E$ in $Q_A \otimes \widehat{E}$, i.e. $W = \begin{pmatrix} I_E \\ 0_{E^{\perp}} \end{pmatrix}$. Defining

$|\widehat{\psi}\rangle\!\langle\widehat{\psi}| = (I \otimes W)|\psi\rangle\!\langle\psi|(I \otimes W^*)$ we see that the objective value remains unchanged,

$$\begin{aligned}\sum_a \mathrm{Tr}\left[(M_a \otimes \frac{S_a + S_a^*}{2})|\widehat{\psi}\rangle\!\langle\widehat{\psi}|\right] &= \sum_a \mathrm{Tr}\left[(M_a \otimes \frac{S_a + S_a^*}{2})(I \otimes W)|\psi\rangle\!\langle\psi|(I \otimes W^*)\right] \\ &= \sum_a \mathrm{Tr}\left[(M_a \otimes \frac{W^* S_a W + W^* S_a^* W}{2})|\psi\rangle\!\langle\psi|\right] \\ &= \sum_a \mathrm{Tr}\left[(M_a \otimes \frac{V_a + V_a^*}{2})|\psi\rangle\!\langle\psi|\right].\end{aligned} \tag{24}$$

Thus we have $\widehat{Q}^{\mathrm{DI}}_{(2)} \geq Q^{\mathrm{DI}}_{(2)}$. However, as the optimizations range over all Hilbert spaces (assuming also infinite dimensional) we have that any feasible point of (23) is trivially a feasible point of (22) and so $\widehat{Q}^{\mathrm{DI}}_{(2)} \leq Q^{\mathrm{DI}}_{(2)}$. Therefore we conclude that $\widehat{Q}^{\mathrm{DI}}_{(2)} = Q^{\mathrm{DI}}_{(2)}$ and we can impose the additional restrictions of (23) when we drop the constraints $V_a + V_a^* \geq 0$.

Unfortunately, the dilation theorem does not immediately apply to the optimization that includes the operator inequalities $V_a + V_a^* \geq 0$ as it need not hold that $S_a + S_a^* \geq 0$ if $V_a + V_a^* \geq 0$. One workaround is to drop these constraints from the optimization, which is was what was done when computing the rate plots from the main text. Alternatively, we can relax the constraint to a moment inequality as $\mathrm{Tr}\left[(V_a + V_a^*)|\psi\rangle\!\langle\psi|\right] \geq 0 \implies \mathrm{Tr}\left[(S_a + S_a^*)|\widehat{\psi}\rangle\!\langle\widehat{\psi}|\right] \geq 0$.

What remains is to consider how this dilation theorem may be used to impose additional constraints on the other conditional entropies $H^{\uparrow}_{(\alpha_k)}$. For simplicity, let us consider the case of $\alpha_k = 4/3$, for the other α_k the procedure remains the same. Recall that,

$$\begin{aligned} Q^{\mathrm{DI}}_{(4/3)} = &\sup_{\{V_{1,a}\}_a, \{V_{2,a}\}_a, \{M_a\}_a, |\psi\rangle\!\langle\psi|, Q_A \otimes E} \sum_a \mathrm{Tr}\left[(M_a \otimes \frac{V_{1,a} + V_{1,a^*}}{2})|\psi\rangle\!\langle\psi|\right] \\ &\text{s.t.} \qquad \sum_a V_{2,a}^* V_{2,a} \leq I_E \\ &\qquad\qquad V_{1,a}^* V_{1,a} \leq \frac{V_{2,a} + V_{2,a}^*}{2} \qquad \text{for all } a \in \mathcal{A}. \end{aligned} \tag{25}$$

Following the previous construction we can define a larger Hilbert space $\widehat{E}$ and some operators $\{S_{2,a}\}_a$ that play the role of $\{V_{2,a}\}$ but satisfy the additional restriction of being coisometries with orthogonal ranges. Unfortunately, we run into similar problems to the ones that we faced with the operator inequalities $V_a + V_a^* \geq 0$ when dilating $H^{\uparrow}_{(2)}$. If we embed $\{V_{1,a}\}$ and $|\psi\rangle\!\langle\psi|$ using the isometry W as before, the objective value remains unchanged but the constraints $V_{1,a}^* V_{1,a} \leq \frac{V_{2,a}+V_{2,a}}{2}$ must be interpreted on the subspace E. This is because $V_{1,a}^* V_{1,a} \leq \frac{V_{2,a}+V_{2,a}^*}{2} \not\Longrightarrow W V_{1,a}^* V_{1,a} W^* \leq \frac{S_{2,a}+S_{2,a}^*}{2}$. To see this note that the left-hand-side of the second inequality has support only on the subspace E but the right-hand-side may have support elsewhere and need not be positive semidefinite a priori.

Again, we can weaken this constraint from an operator inequality to a trace inequality

$$\mathrm{Tr}\left[V_{1,a}^* V_{1,a}|\psi\rangle\!\langle\psi|\right] \leq \mathrm{Tr}\left[\frac{V_{2,a} + V_{2,a}^*}{2}|\psi\rangle\!\langle\psi|\right]. \tag{26}$$

For this weaker constraint, its dilated counterpart $\mathrm{Tr}\left[W V_{1,a}^* V_{1,a} W^* |\widehat{\psi}\rangle\!\langle\widehat{\psi}|\right] \leq \mathrm{Tr}\left[\frac{S_{2,a}+S_{2,a}^*}{2}|\widehat{\psi}\rangle\!\langle\widehat{\psi}|\right]$ does hold true as $\mathrm{Tr}\left[S_{2,a}|\widehat{\psi}\rangle\!\langle\widehat{\psi}|\right] = \mathrm{Tr}\left[V_{2,a}|\psi\rangle\!\langle\psi|\right]$. However, after numerical testing we found that this weaker constraint often lead to much weaker results and so for all of the numerical examples we decided not to add any additional constraints to the optimizations of $H^{\uparrow}_{(4/3)}$.

Sufficient relaxation level to observe ordering

We know for a given cq-state ρ_{AE} that $H^{\uparrow}_{(\alpha_k)}(A|E) \geq H^{\uparrow}_{(\alpha_{k-1})}(A|E) \geq H_{\min}(A|E)$. However, when we perform device-independent optimizations of these quantities we relax the optimization problem to

a semidefinite program via the NPA hierarchy [9]. For a given level of relaxation, the corresponding relaxed problems need not always satisfy this ordering. However, it is possible to find a sufficient level of relaxation such that the ordering holds.

For example, consider the commuting operator version of the min-entropy problem

$$\begin{aligned} -\log \max & \sum_a \mathrm{Tr}\left[M_a W_a |\psi\rangle\langle\psi|\right] \\ \text{s.t.} \quad & \sum_a W_a \leq I \\ & W_a \geq 0 \qquad \text{for all } a \in \mathcal{A} \\ & \sum_a M_a = I \\ & M_a \geq 0 \qquad \text{for all } a \in \mathcal{A} \\ & [M_a, W_b] = 0 \qquad \text{for all } a, b \in \mathcal{A} \end{aligned} \tag{27}$$

and the corresponding problem for $H^{\uparrow}_{(2)}(A|E)$

$$\begin{aligned} -2\log \max & \sum_a \mathrm{Tr}\left[M_a \frac{V_a + V_a^*}{2} |\psi\rangle\langle\psi|\right] \\ \text{s.t.} \quad & \sum_a V_a^* V_a \leq I \\ & V_a + V_a^* \geq 0 \qquad \text{for all } a \in \mathcal{A} \\ & \sum_a M_a = I \\ & M_a \geq 0 \qquad \text{for all } a \in \mathcal{A} \\ & [M_a, V_b^{(*)}] = 0 \qquad \text{for all } a, b \in \mathcal{A}. \end{aligned} \tag{28}$$

By applying an appropriate Naimark dilation to the Hilbert space we may assume that $\{M_a\}$ forms a projective measurement. Note that we could also make this assumption for $\{W_a\}$. However, to then establish ordering we would have to include the additional constraints that were introduced in the previous section. For simplicity we do not consider this but the strategy for enforcing an ordering works in the same manner.

We know from the main text that for an explicit state ρ_{AE}, $H^{\uparrow}_{(2)}(A|E)$ and $H_{\min}(A|E)$ are related by the Cauchy-Schwarz inequality

$$\frac{1}{2}\mathrm{Tr}\left[M_a(V_a + V_a^*)|\psi\rangle\langle\psi|\right] \leq \mathrm{Tr}\left[M_a V_a^* V_a |\psi\rangle\langle\psi|\right]^{1/2}. \tag{29}$$

Now consider a certificate Γ of (28) which has the monomials $\{M_a, M_a V_a\}_a$ in its indexing set. Then as $\Gamma \geq 0$, for each a the submatrix

$$\begin{array}{c} \\ M_a \\ M_a V_a \end{array} \begin{array}{c} \begin{array}{cc} \quad M_a \quad & \quad M_a V_a \end{array} \\ \begin{pmatrix} \mathrm{Tr}\left[M_a|\psi\rangle\langle\psi|\right] & \mathrm{Tr}\left[M_a V_a|\psi\rangle\langle\psi|\right] \\ \mathrm{Tr}\left[M_a V_a^*|\psi\rangle\langle\psi|\right] & \mathrm{Tr}\left[M_a V_a^* V_a|\psi\rangle\langle\psi|\right] \end{pmatrix} \end{array} \tag{30}$$

is positive semidefinite. Summing over a, the fact that each submatrix is PSD implies

$$\begin{pmatrix} \sum_a \mathrm{Tr}\left[M_a|\psi\rangle\langle\psi|\right] & \sum_a \mathrm{Tr}\left[M_a V_a|\psi\rangle\langle\psi|\right] \\ \sum_a \mathrm{Tr}\left[M_a V_a^*|\psi\rangle\langle\psi|\right] & \sum_a \mathrm{Tr}\left[M_a V_a^* V_a|\psi\rangle\langle\psi|\right] \end{pmatrix} \geq 0. \tag{31}$$

By Lemma 2 and the fact that $\sum_a \mathrm{Tr}\left[M_a|\psi\rangle\langle\psi|\right] = 1$ this implies that

$$\begin{aligned}\sum_a \mathrm{Tr}\left[M_a V_a^* V_a |\psi\rangle\langle\psi|\right] &\geq \big(\sum_a \mathrm{Tr}\left[M_a V_a^* |\psi\rangle\langle\psi|\right]\big)\big(\sum_a \mathrm{Tr}\left[M_a V_a |\psi\rangle\langle\psi|\right]\big) \\ &= \big(\sum_a \mathrm{Tr}\left[M_a V_a |\psi\rangle\langle\psi|\right]\big)^2\end{aligned} \tag{32}$$

which is exactly the Cauchy-Schwarz relation. The final line follows from the fact that if Γ is a real symmetric matrix, which we can assume as if Γ is a certificate then so is $(\Gamma + \overline{\Gamma})/2$ (where $\overline{\Gamma}$ denotes the entrywise complex conjugate of Γ), then $\mathrm{Tr}\left[M_a V_a|\psi\rangle\langle\psi|\right] = \mathrm{Tr}\left[M_a V_a^*|\psi\rangle\langle\psi|\right]$. Thus, optimizing over such certificates we will always have

$$\sum_a \mathrm{Tr}\left[M_a \frac{V_a + V_a^*}{2}|\psi\rangle\langle\psi|\right] \leq \left(\sum_a \mathrm{Tr}\left[M_a V_a^* V_a|\psi\rangle\langle\psi|\right]\right)^{1/2}. \tag{33}$$

Now suppose Γ_1 is a certificate for (27) and Γ_2 is a certificate for (28) which implies the Cauchy-Schwarz relation above. Then if for each monomial of the form XW_a in the indexing set of Γ_1 we add a corresponding monomial $XV_a^*V_a$ to the indexing set of Γ_2 we will always have

$$\begin{aligned}\max_{\Gamma_2} \sum_a \mathrm{Tr}\left[M_a \frac{V_a + V_a^*}{2}|\psi\rangle\langle\psi|\right] &\leq \max_{\Gamma_2}\left(\sum_a \mathrm{Tr}\left[M_a V_a^* V_a|\psi\rangle\langle\psi|\right]\right)^{1/2} \\ &\leq \max_{\Gamma_1}\left(\sum_a \mathrm{Tr}\left[M_a W_a|\psi\rangle\langle\psi|\right]\right)^{1/2}.\end{aligned} \tag{34}$$

For example, when computing the plots from the main text we relaxed the $H_{\min}$ computations to the second level of the hierarchy. Then a sufficient relaxation for the $H^{\uparrow}_{(2)}$ computations is the second level of the hierarchy together with monomials $\{M_{a|x}V_c^*V_c\}_{a,x,c} \cup \{N_{b|y}V_c^*V_c\}_{b,y,c}$ where $\{M_{a|x}\}_{a,x}$ are operators representing Alice's measurements and $\{N_{b|y}\}_{b,y}$ are operators representing Bob's measurements.

Let us now consider the case of $H^{\uparrow}_{(4/3)}(A|E)$ from which the general case of $H^{\uparrow}_{(\alpha_k)}(A|E)$ follows readily. For this optimization we have additional operator inequalities

$$V_{1,a}^* V_{1,a} \leq \frac{V_{2,a} + V_{2,a}^*}{2} \tag{35}$$

for each $a \in \mathcal{A}$. Operator inequalities are imposed within the NPA hierarchy via localizing matrices (cf. (20)). That is, we take a collection of monomials $\mathcal{W}_{\mathrm{loc}} = \{X_1, \ldots X_k\}$ indexing a localizing matrix $\Gamma^{\mathrm{loc}} \geq 0$ whose (X_i, X_j) entry corresponds to

$$\mathrm{Tr}\left[X_i^*\left(\frac{V_{2,a} + V_{2,a}^*}{2} - V_{1,a}^* V_{1,a}\right)X_j|\psi\rangle\langle\psi|\right], \tag{36}$$

for each $X_i, X_j \in \mathcal{W}$. If the monomials $\{M_a\}$ corresponding to Alice's measurement operators are included in this localizing set $\mathcal{W}_{\mathrm{loc}}$ then $\Gamma^{\mathrm{loc}} \geq 0$ enforces that

$$\Gamma^{\mathrm{loc}}_{(M_a, M_a)} = \mathrm{Tr}\left[M_a\left(\frac{V_{2,a} + V_{2,a}^*}{2} - V_{1,a}^* V_{1,a}\right)|\psi\rangle\langle\psi|\right] \geq 0. \tag{37}$$

By linearity of the trace this implies that $\mathrm{Tr}\left[M_a \frac{V_{2,a}+V_{2,a}^*}{2}|\psi\rangle\langle\psi|\right] \geq \mathrm{Tr}\left[M_a V_{1,a}^* V_{1,a}|\psi\rangle\langle\psi|\right]$. As in the above example for $H^{\uparrow}_{(2)}(A|E)$, if we add enough monomials to the indexing set of the certificate Γ we can enforce Cauchy-Schwarz relations (cf. (32)). The Cauchy-Schwarz relation allows us to conclude that

$$\max_{\Gamma} \sum_a \mathrm{Tr}\left[M_a \frac{V_{1,a} + V_{1,a}^*}{2}|\psi\rangle\langle\psi|\right] \leq \max_{\Gamma}\left(\sum_a \mathrm{Tr}\left[M_a V_{1,a}^* V_{1,a}|\psi\rangle\langle\psi|\right]\right)^{1/2} \tag{38}$$

and if we have sufficient monomials indexing the localizing matrices we can further conclude that

$$\max_{\Gamma} \left(\sum_a \mathrm{Tr}\left[M_a V_{1,a}^* V_{1,a} |\psi\rangle\langle\psi| \right] \right)^{1/2} \leq \max_{\Gamma} \left(\sum_a \mathrm{Tr}\left[M_a \frac{V_{2,a} + V_{2,a}^*}{2} |\psi\rangle\langle\psi| \right] \right)^{1/2} \tag{39}$$

which is the objective function for $H^{\uparrow}_{(2)}(A|E)$. Note that we can move the max inside the exponentiation as $t \mapsto t^{1/2}$ is monotonic. Furthermore the exponent can be taken outside of the logarithm to cancel with the extra multiplicative factor of 2 that $H^{\uparrow}_{(4/3)}$ has. For general $H^{\uparrow}_{(\alpha_k)}$ this procedure can be repeated, including enough monomials in the certificate to enforce all of the Cauchy-Schwarz relations and for each operator inequality adding enough monomials to its corresponding localizing matrix to enforce the tracial inequalities of the form (37).

Remark 1. It is important that all necessary monomials are included. For example, it is common when certain variables in the optimization form a n-outcome POVM to remove one of them from the indexing set, e.g., defining the final element as $I - M_1 - M_2 - \cdots - M_{n-1}$. However, if this is done for the $\{M_a\}$ that appear in the objective function of $H^{\uparrow}_{(\alpha_k)}(A|E)$ then this will result in suboptimal rates as the relevant Cauchy-Schwarz relations will not be imposed.

From SDPs to min-tradeoff functions

As noted in the main text, solutions to our device-independent optimizations may be combined with the entropy accumulation theorem [11, 12] in order to prove security of the respective device-independent protocols [13, 14]. The entropy accumulation theorem specifies that, under reasonable assumptions, the total smooth min-entropy of a large system can be lower bounded by the total von Neumann entropy of its subsystems minus some correction term that scales sublinearly in the number of subsystems, i.e.

$$H^{\epsilon}_{\min}(A_1^n B_1^n | X_1^n Y_1^n E) > \sum_{i=1}^{n} H(A_i B_i | X_i Y_i E) - O(\sqrt{n}), \tag{40}$$

where $F_1^n = F_1 F_2 \dots F_n$. The total smooth min-entropy characterizes the number of random uniform bits that can be extracted from $A_1^n B_1^n$ and so operationally corresponds to the length of the raw secret key for QKD (before losses due to error correction are taken into account) or the amount of gross uniform randomness acquired in randomness expansion. In order to use the entropy accumulation theorem to prove security of the protocol, one is required to construct min-tradeoff functions. Recall that these are functions which lower bound the quantities $H(A_i B_i | X_i Y_i E)$ in terms of some expected values of some statistical test $C : \mathcal{ABXY} \to \mathcal{C}$, e.g. an expected Bell-inequality violation. In the following we will show how it is possible to extract min-tradeoff functions directly from the solutions to our device-independent optimizations.

Suppose we have a primal SDP of the following form

$$\begin{aligned} p^*(b) := \sup_X \quad & \mathrm{Tr}\left[C\, X \right] \\ \text{s.t.} \quad & \mathrm{Tr}\left[F_i X \right] \geq b_i \qquad \text{for all } i = 1, \dots r \\ & X \geq 0 \end{aligned} \tag{41}$$

where $C, F_1, \dots, F_r$ are real symmetric matrices and $b_i \in \mathbb{R}$. In the context with which we are concerned X would correspond to a moment matrix of the NPA hierarchy and the inequality constraints impose the various constraints of the relaxation as well as the statistical constraints, e.g. a Bell-inequality violation. Note that we can impose equality constraints via two inequality constraints, i.e. $a \geq b$ and $-a \geq -b$ together imply $a = b$. We chose to use the primal form with inequality constraints as this is how we implemented the SDPs, a similar computation could be done for an SDP with equality constraints.

The dual of this optimization problem can be expressed as

$$\begin{aligned} d^*(b) := \inf_{\lambda_i \leq 0} \quad & \sum_i \lambda_i b_i \\ \text{s.t.} \quad & C - \sum_i \lambda_i F_i - Y \leq 0 \\ & Y \leq 0. \end{aligned} \tag{42}$$

Both the primal and the dual programs are parameterized by the constraint vector $b = (b_1, \ldots, b_r)$. We will now show that we can use any feasible point of the dual program parameterized by b to bound the optimal solution to the primal program parameterized by some other constraint vector $\hat{b} \in \mathbb{R}^r$. Let (λ, Y) be a feasible point of (42) when parameterized by the constraint vector b and let $\widehat{X}$ be a feasible point of (41) when parameterized by the constraint vector $\hat{b}$. Then we have

$$\begin{aligned} 0 &\geq \mathrm{Tr}\left[(C - \sum_i \lambda_i F_i - Y)\widehat{X}\right] \\ &\geq \mathrm{Tr}\left[C\widehat{X}\right] - \sum_i \lambda_i \mathrm{Tr}\left[F_i \widehat{X}\right] \\ &\geq \mathrm{Tr}\left[C\widehat{X}\right] - \sum_i \lambda_i \hat{b}_i. \end{aligned} \tag{43}$$

Thus, taking the supremum over all feasible $\widehat{X}$ we have $\sum_i \lambda_i \hat{b}_i \geq p^*(\hat{b})$.

In the context of our device-independent optimizations we only need to vary certain parts of the constraint vector, i.e. the parts that correspond to the values of the statistical test. Therefore we can order the constraint vector such that it partitions into two smaller constraint vectors b_{fix} and b_{var} which are the fixed and varying parts of the full constraint vector respectively. We can also then partition the dual solution vector $\lambda = (\lambda_{\text{fix}}, \lambda_{\text{var}})$ in the same way. Writing $\alpha = \lambda_{\text{fix}} \cdot b_{\text{fix}}$ we have that the dual solution provides us with an affine function $g(\hat{b}) := \alpha + \lambda_{\text{var}} \cdot \hat{b}_{\text{var}}$ which is always an upper bound on the primal program, $g(\hat{b}) \geq p^*(\hat{b})$.

Let us return to the task of constructing min-tradeoff functions. Recall that a statistical test is some function $C : \mathcal{ABXY} \to \mathcal{C}$. Given a distribution $q : \mathcal{C} \to [0, 1]$, we say a strategy $(Q_A, Q_B, E, |\psi\rangle, \{M_{a|x}\}, \{N_{b|y}\})$ is compatible with the statistics q if for all $c \in \mathcal{C}$ we have

$$\sum_{abxy : C(a,b,x,y) = c} \mu(x, y) p(a, b|x, y) = q(c), \tag{44}$$

where μ is some probability distribution on $\mathcal{XY}$. Then a function $f : \mathcal{P}(\mathcal{C}) \to \mathbb{R}$ is a global min-tradeoff function for the statistical test C if it satisfies

$$f(q) \leq \inf_{\Sigma_C(q)} H(AB|XYE) \tag{45}$$

where the infimum is taken over all post-measurement states of all finite-dimensional strategies that are compatible with statistics q. Similarly, we call f a local min-tradeoff function if $f(q) \leq \inf_{\Sigma_C(q)} H(A|XE)$.

Let $p^*_{\text{NPA}}(q)$ be the optimal solution to an NPA relaxation of $Q^{\text{DI}}_{(\alpha_k)}$ (see (22) and (28)) with additional constraints of the form

$$\pm \sum_{abxy : C(a,b,x,y) = c} \mu(x, y) \mathrm{Tr}\left[(M_{a|x} \otimes N_{b|y} \otimes I_E) |\psi\rangle\!\langle\psi|\right] \geq \pm q(c). \tag{46}$$

Then for any $k \in \mathbb{N}$ and some fixed $(x_0, y_0) \in \mathcal{X}\mathcal{Y}$ we have

$$\begin{aligned} \inf_{\Sigma_C(q)} H(AB|XYE) &\geq \inf_{\Sigma_C(q)} \mu(x_0,y_0) H(AB|X=x_0, Y=y_0, E) \\ &= \mu(x_0,y_0) \inf_{\Sigma_C(q)} \frac{\alpha_k}{1-\alpha_k} \log Q^{\mathrm{DI}}_{(\alpha_k)} \\ &\geq \mu(x_0,y_0) \frac{\alpha_k}{1-\alpha_k} \log p^*_{\mathrm{NPA}}(q) \\ &\geq \mu(x_0,y_0) \frac{\alpha_k}{1-\alpha_k} \log(\alpha + \lambda \cdot q). \end{aligned} \tag{47}$$

That is, we can lower bound the von Neumann entropy with an iterated means entropy, solve the relaxed optimization of the iterated means entropy and then extract from the dual solution a lower bounding functional. Many device-independent protocols use a spot-checking procedure wherein the statistical test is performed infrequently and with high probability some fixed inputs (x_0, y_0) are input to the devices. Hence the probability $\mu(x_0, y_0)$ will be close to one. In applications of the EAT the min-tradeoff functions are restricted to be affine functions of the statistics. However, as $-\log(\alpha + \lambda \cdot q)$ is a convex function of q and so one can derive an affine lower bound by taking a first order Taylor expansion of $\mu(x_0, y_0)\frac{\alpha_k}{1-\alpha_k}\log(\alpha + \lambda \cdot q)$. The resulting function can then be used directly with the EAT. For example, we could repeat the analysis of [15], which gave security proofs for randomness expansion using min-tradeoff functions derived from the min-entropy program, using our iterated means entropies. Given the comparisons between the iterated mean entropies and the min-entropy presented in the main text, redoing the security proofs in [15] with the iterated mean entropies would likely give substantial improvements on the finite round rates. For DI-QKD protocols one could look to adapt the analysis of [14], replacing the tradeoff function derived for the CHSH game [16] with tradeoff functions derived from our SDPs.

Additional plots

Results for the bounds on local randomness for 2-input 2-output devices constrained by their full conditional distribution are presented in Figure 1. As explained in the main text, for each detection efficiency we allow ourselves to optimize over some class of two-qubit systems to find a conditional distribution that maximizes the rate. We see a large difference between $H^{\uparrow}_{(2)}(A|E)$ and $H_{\min}(A|E)$. However, like in the corresponding plot for global randomness presented in the main document, we see a negligible improvement on the randomness certified when comparing $H^{\uparrow}_{(4/3)}(A|E)$ and $H^{\uparrow}_{(2)}(A|E)$. Comparing with the analytical bound from [16] and the TSGPL bound from [17], we found our bounds are almost everywhere lower. An exception to this is in the regime of high detection efficiencies where our lower bounds converge to the optimum value of one and so surpass the TSGPL bound.

Another interesting comparison is to see whether $H^{\uparrow}_{(\alpha_k)}(A|E)$ converges to $H(A|E)$ when the devices are constrained only by a CHSH score. In this case we know tight analytical bounds on both $H(A|E)$ [16] and $H_{\min}(A|E)$ [19]. In Figure 2 we include an additional plot that compares lower bounds the local randomness as measured by $H(A|E)$, $H^{\uparrow}_{(8/7)}(A|E)$ and $H_{\min}(A|E)$. Unfortunately, we find in this scenario that our technique gives only a negligible improvement over $H_{\min}$ for the entropies in the family which we could compute.

We suspect that this lack of improvement may be related to evidence that both the Petz $\overline{H}^{\uparrow}_{\alpha}(A|E)$ and geometric $\widehat{H}^{\uparrow}_{\alpha}(A|E)$ may converge slowly in this scenario. For example, we know that $\overline{H}^{\uparrow}_{\alpha}(A|E)$ should converge to $H(A|E)$ as $\alpha \to 1$. However, we can show that when the devices are constrained by an expected CHSH score that $\inf \overline{H}^{\uparrow}_{2}(A|E) = \inf H_{\min}(A|E)$. We provide a proof of these statements in Lemma 1 and Corollary 1. It is also known that for the sandwiched entropies we have $\inf \widetilde{H}^{\downarrow}_{2}(A|E) = \inf H_{\min}(A|E)$ [20]. The fact that these entropies provide no improvement at all over $H_{\min}(A|E)$ is consistent with the results we see in Figure 2.

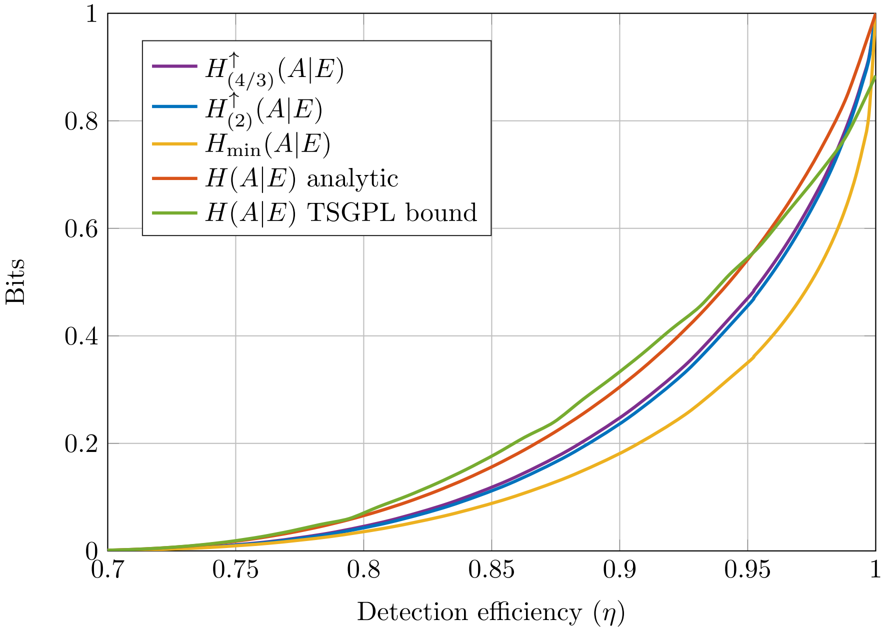

Figure 1: **Local randomness vs. detection efficiency (η) in the 2222-scenario.** We compare lower bounds on different measures of the global randomness produced by 2-input 2-output devices that have some fixed detection efficiency $\eta \in [0.7, 1]$. The curves for $H^{\uparrow}_{(4/3)}(A|E)$, $H^{\uparrow}_{(2)}(A|E)$ and $H_{\min}(A|E)$ were computed numerically, the red curve representing $\inf H(A|E)$ was computed using the analytical expression from [16] and the TSGPL bound uses data from the authors of [17]. The red curve (analytic) was computed by maximizing the CHSH score over two-qubit systems with a fixed η. All other curves constrained the devices to satisfy some fixed probability distribution. For the TSGPL bound this distribution was chosen by maximizing the CHSH score for a fixed η. For the remainder of the curves we optimized our choice of distribution using the method of [18].

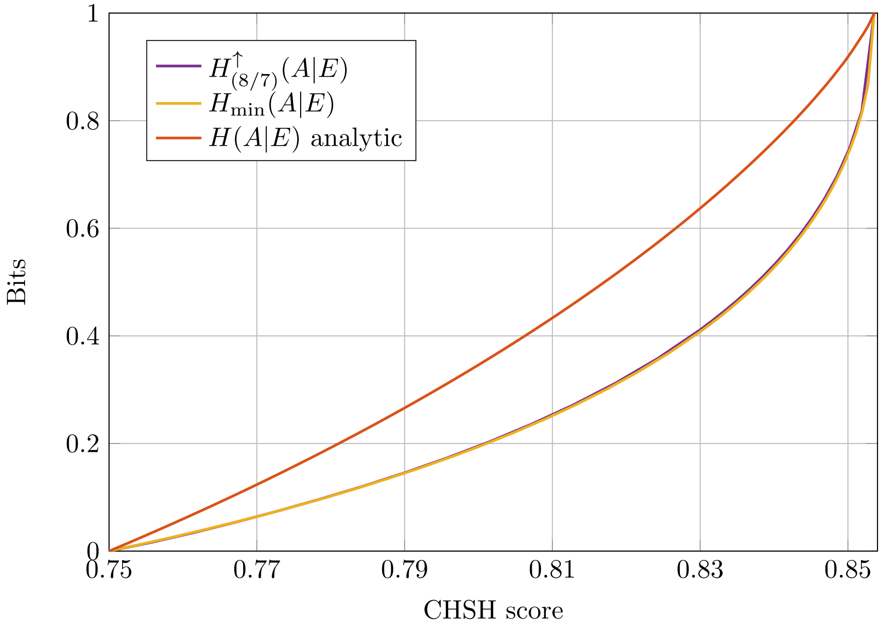

Figure 2: **Local randomness vs. expected CHSH score.** The curve of $H^{\uparrow}_{(8/7)}(A|E)$ was computed numerically, the red curve representing $\inf H(A|E)$ was computed using the analytical expression from [16] and the curve representing $\inf H_{\min}(A|E)$ was computed using the analytical expression from [19].

Proof of Proposition 2

For ease of reading recall that the iterated mean divergences are defined, for $k \in \mathbb{N}$ and $\alpha_k = 1 + \frac{1}{2^k - 1}$ as

$$D_{(\alpha_k)}(\rho\|\sigma) := \frac{1}{\alpha_k - 1} \log Q_{(\alpha_k)}(\rho\|\sigma) \tag{48}$$

where

$$\begin{aligned} Q_{(\alpha_k)}(\rho\|\sigma) := \max_{V_1,\dots,V_k,Z} \quad & \alpha_k \mathrm{Tr}\left[\rho \frac{(V_1 + V_1^*)}{2}\right] - (\alpha_k - 1)\mathrm{Tr}\left[\sigma Z\right] \\ \text{s.t.} \quad & V_1 + V_1^* \geq 0 \\ & \begin{pmatrix} I & V_1 \\ V_1^* & \frac{(V_2+V_2^*)}{2} \end{pmatrix} \geq 0 \quad \begin{pmatrix} I & V_2 \\ V_2^* & \frac{(V_3+V_3^*)}{2} \end{pmatrix} \geq 0 \quad \cdots \quad \begin{pmatrix} I & V_k \\ V_k^* & Z \end{pmatrix} \geq 0. \end{aligned} \tag{49}$$

Before we begin the proof of the proposition we make an observation that we can assume the support of all operators within the optimization is contained within the support of σ, i.e., $\sigma \gg Z$ and $\sigma \gg V_i$ for all $1 \leq i \leq k$. To see this consider the decomposition of the Hilbert space as $\mathcal{H} = \mathrm{supp}(\sigma) \oplus \mathrm{supp}(\sigma)^{\perp}$. With respect to this decomposition we may write the operators in block matrix form as

$$\rho = \begin{pmatrix} \rho(0,0) & 0 \\ 0 & 0 \end{pmatrix}, \quad \sigma = \begin{pmatrix} \sigma(0,0) & 0 \\ 0 & 0 \end{pmatrix}, \quad V_i = \begin{pmatrix} V_i(0,0) & V_i(0,1) \\ V_i(1,0) & V_i(1,1) \end{pmatrix}, \quad Z = \begin{pmatrix} Z(0,0) & Z(0,1) \\ Z^*(0,1) & Z(1,1) \end{pmatrix}. \tag{50}$$

With this form the objective function may be written as

$$\alpha_k \mathrm{Tr}\left[\rho(0,0)\frac{V_1(0,0) + V_1^*(0,0)}{2}\right] - (1 - \alpha_k)\mathrm{Tr}\left[\sigma(0,0)Z(0,0)\right] \tag{51}$$

and so only depends on the restriction of the operators to the subspace $\mathrm{supp}(\sigma)$. Now the positive-semidefinite constraints in (49) may be rewritten as $V_i^* V_i \leq \frac{V_{i+1} + V_{i+1}^*}{2}$ for $1 \leq i \leq k-1$ and $V_k^* V_k \leq Z$.

By direct computation we find that

$$\frac{V_{i+1}+V_{i+1}^*}{2} - V_i^* V_i = \begin{pmatrix} \frac{V_{i+1}(0,0)+V_{i+1}^*(0,0)}{2} - V_i^*(0,0)V_i(0,0) - V_i^*(1,0)V_i^*(1,0) & * \\ * & * \end{pmatrix} \tag{52}$$

and so $\frac{V_{i+1}+V_{i+1}^*}{2} - V_i^*V_i \geq 0 \implies \frac{V_{i+1}(0,0)+V_{i+1}^*(0,0)}{2} - V_i^*(0,0)V_i(0,0) - V_i^*(1,0)V_i(1,0) \geq 0 \implies \frac{V_{i+1}(0,0)+V_{i+1}^*(0,0)}{2} - V_i^*(0,0)V_i(0,0) \geq 0$. The final implication holds because $V_i^*(1,0)V_i(1,0) \geq 0$. Similarly, for the positive semidefinite constraint involving Z we find $Z \geq V_k^*V_k \implies Z(0,0) \geq V_k^*(0,0)V_k(0,0)$. Finally $V_1 + V_1^* \geq 0 \implies V_1(0,0) + V_1^*(0,0) \geq 0$. Thus, denoting the projector onto the subspace $\mathrm{supp}(\sigma)$ by Π, we have that for any feasible point $(V_1, \ldots, V_k, Z)$, the point $(\Pi V_1 \Pi, \ldots, \Pi V_k \Pi, \Pi Z \Pi)$ is also feasible, obtains the same objective value and all operators have their support contained in $\mathrm{supp}(\sigma)$. We therefore assume henceforth that all operators in the optimization have their support contained within $\mathrm{supp}(\sigma)$.

Proposition 2. Let $\rho \in \mathscr{D}(\mathcal{H})$, $\sigma \in \mathscr{P}(\mathcal{H})$ and $k \in \mathbb{N}$. Then the following all hold:

1. (Rescaling)

$$\begin{aligned} Q_{(\alpha_k)}(\rho\|\sigma) = \max_{V_1,\ldots,V_k,Z} \quad & \left(\mathrm{Tr}\left[\rho\frac{(V_1+V_1^*)}{2}\right]\right)^{\alpha_k} \\ \text{s.t.} \quad & \mathrm{Tr}\left[\sigma Z\right] = 1 \\ & V_1 + V_1^* \geq 0 \\ & \begin{pmatrix} I & V_1 \\ V_1^* & \frac{(V_2+V_2^*)}{2} \end{pmatrix} \geq 0 \quad \begin{pmatrix} I & V_2 \\ V_2^* & \frac{(V_3+V_3^*)}{2} \end{pmatrix} \geq 0 \quad \cdots \quad \begin{pmatrix} I & V_k \\ V_k^* & Z \end{pmatrix} \geq 0\,. \end{aligned} \tag{53}$$

2. (Dual formulations) We have

$$\begin{aligned} Q_{(\alpha_k)}(\rho\|\sigma) = \min_{A_1,\ldots,A_k,C_1,\ldots,C_k} \quad & \frac{1}{2^k-1}\sum_{i=1}^k 2^{k-i}\mathrm{Tr}\left[A_i\right] \\ \text{s.t.} \quad & C_1 \geq \rho \\ & \begin{pmatrix} A_1 & C_1 \\ C_1 & C_2 \end{pmatrix} \geq 0 \quad \begin{pmatrix} A_2 & C_2 \\ C_2 & C_3 \end{pmatrix} \geq 0 \quad \cdots \quad \begin{pmatrix} A_k & C_k \\ C_k & \sigma \end{pmatrix} \geq 0\,. \end{aligned} \tag{54}$$

   Or also

$$\begin{aligned} Q_{(\alpha_k)}(\rho\|\sigma) = \min_{A_1,\ldots,A_k,C_1,\ldots,C_k} \quad & \mathrm{Tr}\left[A_1\right] \\ \text{s.t.} \quad & \mathrm{Tr}\left[A_1\right] = \mathrm{Tr}\left[A_2\right] = \cdots = \mathrm{Tr}\left[A_k\right] \\ & C_1 \geq \rho \\ & \begin{pmatrix} A_1 & C_1 \\ C_1 & C_2 \end{pmatrix} \geq 0 \quad \begin{pmatrix} A_2 & C_2 \\ C_2 & C_3 \end{pmatrix} \geq 0 \quad \cdots \quad \begin{pmatrix} A_k & C_k \\ C_k & \sigma \end{pmatrix} \geq 0\,. \end{aligned} \tag{55}$$

   Finally and eponymously

$$\begin{aligned} Q_{(\alpha_k)}(\rho\|\sigma) = \min_{A_1,\ldots,A_k} \quad & \mathrm{Tr}\left[A_1\right] \\ \text{s.t.} \quad & \mathrm{Tr}\left[A_1\right] = \mathrm{Tr}\left[A_2\right] = \cdots = \mathrm{Tr}\left[A_k\right] \\ & \rho \leq A_1\#(A_2\#(\ldots\#(A_k\#\sigma)\ldots)). \end{aligned} \tag{56}$$

3. (Submultiplicativity) Let $\rho_1 \in \mathscr{D}(\mathcal{H}_1)$, $\sigma_1 \in \mathscr{P}(\mathcal{H}_1)$, $\rho_2 \in \mathscr{D}(\mathcal{H}_2)$ and $\sigma_2 \in \mathscr{P}(\mathcal{H}_2)$. Then,

$$D_{(\alpha_k)}(\rho_1 \otimes \rho_2\|\sigma_1 \otimes \sigma_2) \leq D_{(\alpha_k)}(\rho_1\|\sigma_1) + D_{(\alpha_k)}(\rho_2\|\sigma_2)\,. \tag{57}$$

4. (Relation to other Rényi divergences)
$$D^{\mathbb{M}}_{\alpha_k}(\rho\|\sigma) \leq \widetilde{D}_{\alpha_k}(\rho\|\sigma) \leq D_{(\alpha_k)}(\rho\|\sigma) \leq \widehat{D}_{\alpha_k}(\rho\|\sigma) \tag{58}$$

5. (Decreasing in k) For all $k \geq 2$,
$$D_{(\alpha_k)}(\rho\|\sigma) \leq D_{(\alpha_{k-1})}(\rho\|\sigma). \tag{59}$$

6. (Data processing) Let $\mathcal{K}$ be another Hilbert space and let $\mathcal{E} : \mathscr{L}(\mathcal{H}) \to \mathscr{L}(\mathcal{K})$ be a CPTP map, then
$$D_{(\alpha_k)}(\rho\|\sigma) \geq D_{(\alpha_k)}(\mathcal{E}(\rho)\|\mathcal{E}(\sigma)). \tag{60}$$

7. (Reduction to classical divergence) If $[\rho, \sigma] = 0$ then
$$D_{(\alpha_k)}(\rho\|\sigma) = \frac{1}{\alpha_k - 1} \log \mathrm{Tr}\left[\rho^{\alpha_k}\sigma^{1-\alpha_k}\right]. \tag{61}$$

Proof

Property 1. Rescaling

For any $\beta > 0$ we have $\begin{pmatrix} A & B \\ B^* & C \end{pmatrix} \geq 0 \iff \begin{pmatrix} A & \beta B \\ \beta B^* & \beta^2 C \end{pmatrix} \geq 0$. It follows then that for any feasible point $(V_1, \ldots, V_k, Z)$ of (49), $(\beta V_1, \beta^2 V_2, \ldots, \beta^{2^{k-1}} V_k, \beta^{2^k} Z)$ is another feasible point. This new feasible point has an objective value $\alpha_k \beta \mathrm{Tr}\left[\rho\frac{(V_1+V_1^*)}{2}\right] - (\alpha_k - 1)\beta^{2^k}\mathrm{Tr}\left[\sigma Z\right]$. We may also assume that $\mathrm{Tr}\left[\rho\frac{(V_1+V_1^*)}{2}\right] \geq 0$ and $\mathrm{Tr}\left[\sigma Z\right] > 0$ by the following argument. As $V_1 + V_1^* \geq 0$ we have $\mathrm{Tr}\left[\rho(V_1 + V_1^*)\right] \geq 0$. Furthermore, as $Z \geq 0$ and $Z \ll \sigma$ we have $\mathrm{Tr}\left[\sigma Z\right] = 0 \iff Z = 0$. However if $Z = 0$ then it follows from the other constraints that we must also have $V_1 = V_2 = \cdots = V_k = 0$ and in turn the objective value is trivially 0. We also have that for any $c > 0$, the point $(cI, 2c^2 I, \ldots, 2^{2^{k-1}-1}c^{2^{k-1}} I, 2^{2^k-1}c^{2^k} I)$ is feasible with an objective value $\alpha_k c \mathrm{Tr}\left[\rho\right] - (\alpha_k - 1)2^{2^k-1}c^{2^k}\mathrm{Tr}\left[\sigma\right]$. Rearranging we find that we have a strictly positive objective value when we choose $c < (2^{1-2^k}\frac{\alpha_k}{\alpha_k-1}\frac{\mathrm{Tr}[\rho]}{\mathrm{Tr}[\sigma]})^{\frac{1}{2^k-1}}$. Thus the choice of $Z = 0$ is always suboptimal and we may also assume that $\mathrm{Tr}\left[\sigma Z\right] > 0$. So with $\mathrm{Tr}\left[\rho\frac{(V_1+V_1^*)}{2}\right] \geq 0$ and $\mathrm{Tr}\left[\sigma Z\right] > 0$ we may maximize over the choice of $\beta > 0$ and we find a unique maximum occurring at
$$\beta^* = \left(\frac{\alpha_k}{2^k(\alpha_k - 1)}\frac{\mathrm{Tr}\left[\rho\frac{(V_1+V_1^*)}{2}\right]}{\mathrm{Tr}\left[\sigma Z\right]}\right)^{\frac{1}{2^k-1}}. \tag{62}$$

For this choice of β the objective function simplifies to
$$\frac{\mathrm{Tr}\left[\rho\frac{(V_1+V_1^*)}{2}\right]^{\alpha_k}}{\mathrm{Tr}\left[\sigma Z\right]^{\frac{1}{2^k-1}}}. \tag{63}$$

Note that after this rewriting, rescaling the operators as before with some $\beta > 0$ does not change the objective value. Thus, we are free to rescale the operators so that $\mathrm{Tr}\left[\sigma Z\right] = 1$. Therefore we can rewrite the optimization as
$$\begin{aligned} Q_{(\alpha_k)}(\rho\|\sigma) = \max_{V_1,\ldots,V_k,Z} \quad & \mathrm{Tr}\left[\rho\frac{(V_1+V_1^*)}{2}\right]^{\alpha_k} \\ \text{s.t.} \quad & \mathrm{Tr}\left[\sigma Z\right] = 1 \\ & V_1 + V_1^* \geq 0 \\ & \begin{pmatrix} I & V_1 \\ V_1^* & \frac{(V_2+V_2^*)}{2} \end{pmatrix} \geq 0 \quad \begin{pmatrix} I & V_2 \\ V_2^* & \frac{(V_3+V_3^*)}{2} \end{pmatrix} \geq 0 \quad \cdots \quad \begin{pmatrix} I & V_k \\ V_k^* & Z \end{pmatrix} \geq 0. \end{aligned} \tag{64}$$

Property 2a. Dual form (a)
We start by establishing the following dual form, which is not included in the statement for brevity:

$$\begin{aligned}Q_{(\alpha_k)}(\rho\|\sigma) = \min_{A_1,\dots,A_k,C_1,\dots,C_k} &\sum_{i=1}^{k} \mathrm{Tr}\left[A_i\right] \\ \text{s.t.}\quad & C_1 \geq \rho \\ &\begin{pmatrix} A_1 & \frac{\alpha_k}{2}C_1 \\ \frac{\alpha_k}{2}C_1 & C_2\end{pmatrix} \geq 0 \quad \begin{pmatrix} A_2 & \frac{C_2}{2} \\ \frac{C_2}{2} & C_3\end{pmatrix} \geq 0 \quad \cdots \quad \begin{pmatrix} A_k & \frac{C_k}{2} \\ \frac{C_k}{2} & \frac{1}{2^k-1}\sigma\end{pmatrix} \geq 0\,.\end{aligned} \tag{65}$$

Introducing the dual variables $\begin{pmatrix} A_i & B_i \\ B_i^* & C_{i+1}\end{pmatrix}$ for $1 \leq i \leq k$ for the positive-semidefinite constraints and the dual variable C_1 for the constraint $V_1 + V_1^* \geq 0$ we can write the Lagrangian of the problem (49) as

$$\begin{aligned}L &= \alpha_k \mathrm{Tr}\left[\rho\frac{(V_1+V_1^*)}{2}\right] - (\alpha_k - 1)\mathrm{Tr}\left[\sigma Z\right] + \mathrm{Tr}\left[(V_1+V_1^*)C_1\right] \\ &\quad + \mathrm{Tr}\left[A_1 + B_1 V_1^* + B_1^* V_1 + C_2(V_2+V_2^*)/2\right] + \cdots + \mathrm{Tr}\left[A_k + B_k V_1^* + B_k^* V_1 + C_{k+1}Z\right] \\ &= \sum_{i=1}^{k}\mathrm{Tr}\left[A_i\right] + \mathrm{Tr}\left[V_1(\tfrac{\alpha_k}{2}\rho + C_1 + B_1^*) + V_1^*(\tfrac{\alpha_k}{2}\rho + C_1 + B_1)\right] + \cdots + \mathrm{Tr}\left[V_k(\tfrac{1}{2}C_k + B_k^*) + V_k^*(\tfrac{1}{2}C_k + B_k)\right] \\ &\quad + \mathrm{Tr}\left[Z(\tfrac{1}{2}C_{k+1} - (\alpha_k - 1)\sigma)\right] \\ &= \sum_{i=1}^{k}\mathrm{Tr}\left[A_i\right] + 2\mathscr{R}\left(\mathrm{Tr}\left[V_1(\tfrac{\alpha_k}{2}\rho + C_1 + B_1^*)\right]\right) + \cdots + 2\mathscr{R}\left(\mathrm{Tr}\left[V_k(\tfrac{1}{2}C_k + B_k^*)\right]\right) + \mathrm{Tr}\left[Z(\tfrac{1}{2}C_{k+1} - (\alpha_k-1)\sigma)\right]\end{aligned} \tag{66}$$

where for the third equality we used the identity $\mathrm{Tr}\left[X + X^*\right] = 2\mathscr{R}(\mathrm{Tr}\left[X\right])$. Now if we take a maximization over the variables $V_1, \dots, V_k$ and Z, we find that the Lagrangian is finite only if $C_1 + \frac{\alpha_k}{2}\rho + B_1^* = 0$, $B_i = -\frac{1}{2}C_{i-1}$ for $2 \leq i \leq k$ and $C_k = (\alpha_k - 1)\sigma$. Note that the condition $C_1 + \frac{\alpha_k}{2}\rho + B_1^* = 0$ can be rewritten as $-B_1^* \geq \frac{\alpha_k}{2}\rho$ as C_1 does not appear elsewhere. We relabel $-B_1^*$ to $\frac{\alpha_k}{2}C_1$. Also, note that it follows from Lemma 2 that $\begin{pmatrix} A & -B \\ -B^* & C\end{pmatrix} \geq 0 \iff \begin{pmatrix} A & B \\ B^* & C\end{pmatrix} \geq 0$. Therefore we can write the dual problem as

$$\begin{aligned}Q_{(\alpha_k)}(\rho\|\sigma) = \min_{A_1,\dots,A_k,C_1,\dots,C_k} &\sum_{i=1}^{k} \mathrm{Tr}\left[A_i\right] \\ \text{s.t.}\quad & C_1 \geq \rho \\ &\begin{pmatrix} A_1 & \frac{\alpha_k}{2}C_1 \\ \frac{\alpha_k}{2}C_1 & C_2\end{pmatrix} \geq 0 \quad \begin{pmatrix} A_2 & \frac{C_2}{2} \\ \frac{C_2}{2} & C_3\end{pmatrix} \geq 0 \quad \cdots \quad \begin{pmatrix} A_k & \frac{C_k}{2} \\ \frac{C_k}{2} & (\alpha_k - 1)\sigma\end{pmatrix} \geq 0\,.\end{aligned} \tag{67}$$

It remains to show that we have strong duality. In order to show this we observe that for any $c > 0$ the assignment $V_1 = cI$, $V_2 = 2c^2 I, \dots, V_k = 2^{2^{k-1}-1}c^{2^{k-1}}I$ and $Z = 2^{2^k-1}c^{2^k}I$ constitutes a strictly feasible point of the primal program. In the dual problem, for $2 \leq i \leq k-1$ the constraints $\begin{pmatrix} A_i & \frac{1}{2}C_i \\ \frac{1}{2}C_i & C_{i+1}\end{pmatrix} \geq 0$ have a strictly feasible assignment $C_i = C_{i+1} = 2I$ and $A_i = \frac{c}{2}I$ for any $c > 1$. Then the assignment $A_1 = c\frac{\alpha_k^2}{2}$ and $C_1 = 2I$ satisfies the first positive semidefinite constraint and $C_1 \geq \rho$ strictly. Now recall that we may assume that we work in the subspace $\mathrm{supp}(\sigma)$ and so we have $\sigma > 0$. Therefore, the assignment $A_k = \frac{c}{(\alpha_k - 1)}\sigma^{-1}$ satisfies the final constraint strictly. As we have demonstrated strictly feasible points to both the primal and the dual problems, it follows that we have strong duality.

Property 2b. Dual form (b)

Firstly, note that it follows from Lemma 2 that for any $\beta > 0$ we have $\begin{pmatrix} A & \beta B \\ \beta B^* & C \end{pmatrix} \geq 0 \iff \begin{pmatrix} \frac{1}{\beta}A & B \\ B^* & \frac{1}{\beta}C \end{pmatrix} \geq 0$. Then we can rewrite the block matrix constraints of the dual problem (67) as

$$\begin{pmatrix} \frac{2}{\alpha_k}A_1 & C_1 \\ C_1 & \frac{2}{\alpha_k}C_2 \end{pmatrix} \geq 0 \qquad \begin{pmatrix} \frac{4}{\alpha_k}A_2 & \frac{4}{\alpha_k}\frac{1}{2}C_2 \\ \frac{4}{\alpha_k}\frac{1}{2}C_2 & \frac{4}{\alpha_k}C_3 \end{pmatrix} \geq 0 \qquad \cdots \qquad \begin{pmatrix} \frac{2^k}{\alpha_k}A_k & \frac{2^k}{\alpha_k}\frac{1}{2}C_k \\ \frac{2^k}{\alpha_k}\frac{1}{2}C_k & \frac{2^k}{\alpha_k}(\alpha_k - 1)\sigma \end{pmatrix} \geq 0. \tag{68}$$

Making the change of variables $\widehat{A}_i = \frac{2^i}{\alpha_k}A_i$ and $\widehat{C}_i = \frac{2^i}{\alpha_k}C_i$ for $2 \leq i \leq k$, we find that the dual program (67) is equivalent to

$$\begin{aligned} &\min_{A_1,\ldots,A_k,C_1,\ldots,C_k} \frac{1}{2^k - 1}\sum_{i=1}^{k} 2^{k-i}\mathrm{Tr}\left[A_i\right] \\ &\text{s.t.} \quad C_1 \geq \rho \\ &\begin{pmatrix} A_1 & C_1 \\ C_1 & C_2 \end{pmatrix} \geq 0 \quad \begin{pmatrix} A_2 & C_2 \\ C_2 & C_3 \end{pmatrix} \geq 0 \quad \cdots \quad \begin{pmatrix} A_k & C_k \\ C_k & \sigma \end{pmatrix} \geq 0\,, \end{aligned} \tag{69}$$

where we also used the fact that the coefficient of σ simplifies as $\frac{2^k}{\alpha_k}(\alpha_k - 1) = 1$.

Property 2c. Dual form (c)

We now derive the third dual form from the second dual form (69). Firstly, let $\gamma_1 > 0$ and note that it follows from Lemma 2 that for any feasible point $(A_1, \ldots, A_k, C_1, \ldots, C_k)$ of (69),

$$(\gamma_1 A_1, \tfrac{1}{\gamma_1^2}A_2, A_3, \ldots, A_k, C_1, \tfrac{1}{\gamma_1}C_2, \ldots, C_k) \tag{70}$$

is also a feasible point. By setting $\gamma_1 = \left(\frac{\mathrm{Tr}[A_2]}{\mathrm{Tr}[A_1]}\right)^{1/3}$ we have $\mathrm{Tr}\left[\gamma_1 A_1\right] = \mathrm{Tr}\left[\frac{1}{\gamma_1}A_2\right]$. Furthermore, we have for this choice of γ_1 that

$$\begin{aligned} 2\mathrm{Tr}\left[\gamma_1 A_1\right] + \mathrm{Tr}\left[\tfrac{1}{\gamma_1}A_2\right] &= 3\mathrm{Tr}\left[A_1\right]^{2/3}\mathrm{Tr}\left[A_2\right]^{1/3} \\ &\leq 2\mathrm{Tr}\left[A_1\right] + \mathrm{Tr}\left[A_2\right], \end{aligned} \tag{71}$$

where the second line follows from the arithmetic-geometric mean inequality. This shows that for any feasible point we can transform it to another feasible point such that $\mathrm{Tr}\left[A_1\right] = \mathrm{Tr}\left[A_2\right]$ and the objective value does not increase under the transformation.

We shall now demonstrate that we can inductively transform any feasible point into another such that the objective value does not increase and the transformed point satisfies $\mathrm{Tr}\left[A_1\right] = \mathrm{Tr}\left[A_2\right] = \cdots = \mathrm{Tr}\left[A_k\right]$. Suppose we have a feasible point $(A_1, \ldots A_k, C_1, \ldots, C_k)$ such that $\mathrm{Tr}\left[A_1\right] = \mathrm{Tr}\left[A_2\right] = \cdots = \mathrm{Tr}\left[A_{i-1}\right]$ for some $2 \leq i \leq k$. Then by Lemma 2 the point

$$(\gamma_i A_1, \gamma_i A_2, \ldots, \gamma_i A_{i-1}, \gamma_i^{-2(2^i-1)}A_i, A_{i+1}, \ldots, A_k, C_1, \gamma_i^{-1}C_2, \gamma_i^{-3}C_3, \ldots, \gamma_i^{-(2^i-1)}C_i, C_{i+1}, \ldots, C_k) \tag{72}$$

is also feasible. By setting $\gamma_i = \left(\frac{\mathrm{Tr}[A_i]}{\mathrm{Tr}[A_1]}\right)^{\frac{1}{2^{i+1}-1}}$ we get $\mathrm{Tr}\left[\gamma_i A_1\right] = \mathrm{Tr}\left[\gamma_i^{-2(2^i-1)}A_i\right]$. Furthermore, for this choice of γ_i we have

$$\begin{aligned} 2^i\mathrm{Tr}\left[\gamma_i A_1\right] + 2^{i-1}\mathrm{Tr}\left[\gamma_i A_2\right] + \cdots + 2\mathrm{Tr}\left[\gamma_i A_{i-1}\right] + \mathrm{Tr}\left[\gamma_i^{-2(2^i-1)}A_i\right] &= 2(2^i - 1)\mathrm{Tr}\left[\gamma_i A_1\right] + \mathrm{Tr}\left[\gamma_i^{-2(2^i-1)}A_i\right] \\ &= (2^{i+1} - 1)\mathrm{Tr}\left[A_1\right]^{1-\frac{1}{2^{i+1}-1}}\mathrm{Tr}\left[A_i\right]^{\frac{1}{2^{i+1}-1}} \\ &\leq 2(2^i - 1)\mathrm{Tr}\left[A_1\right] + \mathrm{Tr}\left[A_i\right] \\ &= 2^i\mathrm{Tr}\left[A_1\right] + 2^{i-1}\mathrm{Tr}\left[A_2\right] + \cdots + \mathrm{Tr}\left[A_i\right], \end{aligned} \tag{73}$$

where on the first line we used $\mathrm{Tr}[A_1] = \mathrm{Tr}[A_2] = \cdots = \mathrm{Tr}[A_{i-1}]$, the second line we substituted in our choice of γ_i and the third line is another application of the arithmetic-geometric mean inequality. This shows that the objective value of the transformed point is no larger than that of the original point. It then follows by induction that we can transform any feasible point of (69) into one which satisfies $\mathrm{Tr}[A_1] = \mathrm{Tr}[A_2] = \cdots = \mathrm{Tr}[A_k]$ without increasing the objective value. Finally, noting that $\frac{1}{2^k-1}\sum_i 2^{k-i}\mathrm{Tr}[A_1] = \mathrm{Tr}[A_1]$ we find that we can rewrite (69) as

$$\begin{aligned} \min_{A_1,\ldots,A_k,C_1,\ldots,C_k} \quad & \mathrm{Tr}[A_1] \\ \text{s.t.} \quad & \mathrm{Tr}[A_1] = \mathrm{Tr}[A_2] = \cdots = \mathrm{Tr}[A_k] \\ & C_1 \geq \rho \\ & \begin{pmatrix} A_1 & C_1 \\ C_1 & C_2 \end{pmatrix} \geq 0 \quad \begin{pmatrix} A_2 & C_2 \\ C_2 & C_3 \end{pmatrix} \geq 0 \quad \cdots \quad \begin{pmatrix} A_k & C_k \\ C_k & \sigma \end{pmatrix} \geq 0 \,, \end{aligned} \tag{74}$$

Property 2d. Dual form (d) We derive the final dual form from the third dual form (74) – an alternative dual form could be derived by starting at (69). Consider any feasible point $(A_1, \ldots A_k, C_1, \ldots C_k)$ of (74). By Lemma 3 we know that

$$\begin{pmatrix} A_i & C_i \\ C_i & C_{i+1} \end{pmatrix} \geq 0 \implies C_i \leq A_i \# C_{i+1}. \tag{75}$$

Therefore the block matrix constraints of (74) imply the operator inequalities

$$C_1 \leq A_1 \# C_2 \quad C_2 \leq A_2 \# C_3 \quad \ldots \quad C_k \leq A_k \# \sigma. \tag{76}$$

Using the fact that if $C \leq D$ then $A\#C \leq A\#D$, we can combine these inequalities together with $\rho \leq C_1$ to conclude that any feasible point of (74) is also a feasible point of the optimization problem

$$\begin{aligned} \min_{A_1,\ldots,A_k} \quad & \mathrm{Tr}[A_1] \\ \text{s.t.} \quad & \mathrm{Tr}[A_1] = \mathrm{Tr}[A_2] = \cdots = \mathrm{Tr}[A_k] \\ & \rho \leq A_1 \#(A_2 \#(\ldots \#(A_k \# \sigma)\ldots)). \end{aligned} \tag{77}$$

Moreover, the objective value remains unchanged. Now consider a feasible point $(A_1, \ldots A_k)$ of (77). As $\begin{pmatrix} A & A\#B \\ A\#B & B \end{pmatrix} \geq 0$ it follows that by choosing $C_i = A_i \# A_{i+1} \ldots \# A_k \# \sigma$ for each $i = 1, \ldots, k$ that $(A_1, \ldots, A_k, C_1, \ldots, C_k)$ is a feasible point of (74) with the same objective value. Therefore (74) and (77) are equal.

Property 3. Submultiplicativity
Let $(A_1, \ldots, A_k, C_1, \ldots, C_k)$ be the optimal point of the optimization (74) for the parameter pair (ρ, σ) and let $(\widehat{A}_1, \ldots, \widehat{A}_k, \widehat{C}_1, \ldots, \widehat{C}_k)$ be the optimal point of (74) for the parameter pair $(\widehat{\rho}, \widehat{\sigma})$. Then $(A_1 \otimes \widehat{A}_1, \ldots, A_k \otimes \widehat{A}_k, C_1 \otimes \widehat{C}_1, \ldots, C_k \otimes \widehat{C}_k)$ is a feasible point of (74) for the pair $(\rho \otimes \widehat{\rho}, \sigma \otimes \widehat{\sigma})$. Moreover, we then have

$$\begin{aligned} Q_{(\alpha_k)}(\rho \otimes \widehat{\rho} \| \sigma \otimes \widehat{\sigma}) &\leq \mathrm{Tr}\left[A_1 \otimes \widehat{A}_1\right] \\ &= \mathrm{Tr}[A_1]\,\mathrm{Tr}\left[\widehat{A}_1\right] \\ &= Q_{(\alpha_k)}(\rho\|\sigma) Q_{(\alpha_k)}(\widehat{\rho}\|\widehat{\sigma}), \end{aligned} \tag{78}$$

and so $D_{(\alpha_k)}(\rho \otimes \widehat{\rho} \| \sigma \otimes \widehat{\sigma}) \leq D_{(\alpha_k)}(\rho\|\sigma) + D_{(\alpha_k)}(\widehat{\rho}\|\widehat{\sigma})$.

Property 4. Relation to other Rényi divergences

Recall that $D^{\mathbb{M}}_{\alpha_k}(\rho\|\sigma) = \frac{1}{\alpha_k-1}\log\max_{\omega>0}\alpha_k \mathrm{Tr}\left[\rho\omega\right] + (1-\alpha_k)\mathrm{Tr}\left[\sigma\omega^{2^k}\right]$. Any $\omega > 0$ defines a feasible choice $V_i = \omega^{2^{i-1}}$ and $Z = \omega^{2^k}$. This gives us immediately $D_{(\alpha_k)}(\rho\|\sigma) \geq D^{\mathbb{M}}_{\alpha_k}(\rho\|\sigma)$. Then by submultiplicativity, for any integer $n \geq 1$,

$$\begin{aligned} D_{(\alpha_k)}(\rho\|\sigma) &\geq \frac{1}{n} D_{(\alpha_k)}(\rho^{\otimes n}\|\sigma^{\otimes n}) \\ &\geq \frac{1}{n} D^{\mathbb{M}}_{\alpha_k}(\rho^{\otimes n}\|\sigma^{\otimes n}) \ . \end{aligned} \tag{79}$$

Taking the limit as $n \to \infty$, we get the sandwiched Rényi divergence and so $D_{(\alpha_k)}(\rho\|\sigma) \geq \widetilde{D}_{\alpha_k}(\rho\|\sigma)$ [21].

Property 5. Decreasing in k
To show the fact that $D_{(\alpha_k)}$ is decreasing in k, we write using the Cauchy-Schwarz inequality and the fact that $\mathrm{Tr}\left[\rho\right] = 1$,

$$\begin{aligned} D_{(\alpha_k)}(\rho\|\sigma) &= 2^k \log \max_{V_1,\ldots,V_k,Z} \mathrm{Tr}\left[\rho(V_1+V_1^*)/2\right] \\ &\leq 2^k \log \max_{V_1,\ldots,V_k,Z} \sqrt{\mathrm{Tr}\left[\rho V_1^* V_1\right]} \\ &\leq 2^k \log \max_{V_2,\ldots,V_k,Z} \sqrt{\mathrm{Tr}\left[\rho(V_2+V_2^*)/2\right]} \\ &= 2^{k-1} \log \max_{V_2,\ldots,V_k,Z} \mathrm{Tr}\left[\rho(V_2+V_2^*)/2\right] \\ &= D_{(\alpha_{k-1})}(\rho\|\sigma) \end{aligned} \tag{80}$$

where the third line follows from the operator inequality constraint $V_1^*V_1 \leq \frac{V_2+V_2^*}{2}$.

Property 6. Data processing
Let $\mathcal{E}^\dagger$ be the adjoint channel of some CPTP map $\mathcal{E} : \mathscr{L}(A) \to \mathscr{L}(B)$. Note that $\mathcal{E}^\dagger$ is unital and completely positive. Now consider the optimization

$$\begin{aligned} q = \max_{W_1,\ldots,W_k,Y} &\left(\mathrm{Tr}\left[\rho\frac{(\mathcal{E}^\dagger(W_1)+\mathcal{E}^\dagger(W_1)^*)}{2}\right]\right)^{\alpha_k} \\ \text{s.t.} \quad & \mathrm{Tr}\left[\sigma\mathcal{E}^\dagger(Y)\right] = 1 \\ & \mathcal{E}^\dagger(W_1)+\mathcal{E}^\dagger(W_1)^* \geq 0 \\ & \begin{pmatrix} I & \mathcal{E}^\dagger(W_1) \\ \mathcal{E}^\dagger(W_1)^* & \frac{(\mathcal{E}^\dagger(W_2)+\mathcal{E}^\dagger(W_2)^*)}{2}\end{pmatrix} \geq 0 \quad \begin{pmatrix} I & \mathcal{E}^\dagger(W_2) \\ \mathcal{E}^\dagger(W_2)^* & \frac{(\mathcal{E}^\dagger(W_3)+\mathcal{E}^\dagger(W_3)^*)}{2}\end{pmatrix} \geq 0 \quad \cdots \quad \begin{pmatrix} I & \mathcal{E}^\dagger(W_k) \\ \mathcal{E}^\dagger(W_k)^* & \mathcal{E}^\dagger(Y)\end{pmatrix} \geq 0 \ , \end{aligned} \tag{81}$$

where the optimization is over linear operators on B. Identifying $V_i = \mathcal{E}^\dagger(W_i)$ and $Z = \mathcal{E}^\dagger(Y)$ we see that every feasible point for the above optimization defines a feasible point for the optimization $Q_{(\alpha_k)}(\rho\|\sigma)$ with the same objective value. Therefore we must have $Q_{(\alpha_k)}(\rho\|\sigma) \geq q$. Now as $\mathcal{E}^\dagger$ is completely positive it also preserves adjoints, i.e., $\mathcal{E}^\dagger(W^*) = \mathcal{E}^\dagger(W)^*$. Therefore, using the fact that $\mathcal{E}^\dagger$ is also unital, we can rewrite q as

$$\begin{aligned} q = \max_{W_1,\ldots,W_k,Y} &\left(\mathrm{Tr}\left[\mathcal{E}(\rho)\frac{(W_1+W_1^*)}{2}\right]\right)^{\alpha_k} \\ \text{s.t.} \quad & \mathrm{Tr}\left[\mathcal{E}(\sigma)Y\right] = 1, \\ & \mathcal{E}^\dagger(W_1+W_1^*) \geq 0 \\ & (\mathcal{I}_2\otimes\mathcal{E}^\dagger)\begin{pmatrix} I & W_1 \\ W_1^* & \frac{(W_2+W_2^*)}{2}\end{pmatrix} \geq 0 \quad (\mathcal{I}_2\otimes\mathcal{E}^\dagger)\begin{pmatrix} I & W_2 \\ W_2^* & \frac{(W_3+W_3^*)}{2}\end{pmatrix} \geq 0 \quad \cdots \quad (\mathcal{I}_2\otimes\mathcal{E}^\dagger)\begin{pmatrix} I & W_k \\ W_k^* & Y\end{pmatrix} \geq 0 \ . \end{aligned} \tag{82}$$

Writing

$$
\begin{aligned}
Q_{(\alpha_k)}(\mathcal{E}(\rho)\|\mathcal{E}(\sigma)) = & \max_{W_1,\ldots,W_k,Y} \left(\mathrm{Tr}\left[\mathcal{E}(\rho) \frac{(W_1+W_1^*)}{2} \right] \right)^{\alpha_k} \\
& \text{s.t.} \quad \mathrm{Tr}\left[\mathcal{E}(\sigma) Y\right] = 1, \\
& \quad W_1 + W_1^* \geq 0 \\
& \begin{pmatrix} I & W_1 \\ W_1^* & \frac{(W_2+W_2^*)}{2} \end{pmatrix} \geq 0 \quad \begin{pmatrix} I & W_2 \\ W_2^* & \frac{(W_3+W_3^*)}{2} \end{pmatrix} \geq 0 \quad \cdots \quad \begin{pmatrix} I & W_k \\ W_k^* & Y \end{pmatrix} \geq 0\,,
\end{aligned} \tag{83}
$$

we see that we must also have $q \geq Q_{(\alpha_k)}(\mathcal{E}(\rho)\|\mathcal{E}(\sigma))$ as they have the same objective function but each feasible point of the latter is a feasible point of the former as $\mathcal{E}^\dagger$ is completely positive. Hence, we have $Q_{(\alpha_k)}(\rho\|\sigma) \geq q \geq Q_{(\alpha_k)}(\mathcal{E}(\rho)\|\mathcal{E}(\sigma))$ and as $\frac{1}{\alpha_k - 1}\log(\cdot)$ is monotonically increasing for all $k \in \mathbb{N}$ the result follows.

Property 7. Reduction to classical divergence
If $[\rho, \sigma] = 0$ then there exists a common eigenbasis of ρ and σ, i.e. there exists an orthonormal basis $\{|x\rangle\}$ such that $\rho = \sum_x p_x |x\rangle\langle x|$ and $\sigma = \sum_x q_x |x\rangle\langle x|$ with $p_x, q_x \geq 0$ and $\sum_x p_x = \sum_x q_x = 1$. Let $\mathcal{P} : \mathscr{L}(\mathcal{H}) \to \mathscr{L}(\mathcal{H})$ be the pinching map

$$
\mathcal{P}(A) = \sum_x |x\rangle\langle x| A |x\rangle\langle x| \tag{84}
$$

defined by this common eigenbasis. Now consider any feasible point $(A_1, \ldots A_k, C_1, \ldots, C_k)$ of the dual problem (69). As the pinching map $\mathcal{P}$ is completely positive, $\rho = \mathcal{P}(\rho)$ and $\sigma = \mathcal{P}(\sigma)$, it follows that $(\mathcal{P}(A_1), \ldots, \mathcal{P}(A_k), \mathcal{P}(C_1), \ldots, \mathcal{P}(C_k))$ is another feasible point of the dual problem. Moreover, this new feasible point has the same objective value as the original point. Therefore, when ρ and σ commute we may assume that all variables in the optimization also commute.

Now we know that [1, Proposition 3.3.4]

$$
\begin{pmatrix} A_1 & C_1 \\ C_1 & C_2 \end{pmatrix} \geq 0 \implies C_1 \leq A_1 \# C_2 = A_1^{1/2} C_2^{1/2} \tag{85}
$$

where the final equality holds as all operators are assumed to commute. Similarly, we have

$$
\begin{pmatrix} A_2 & C_2 \\ C_2 & C_3 \end{pmatrix} \geq 0 \implies C_2 \leq A_2 \# C_3 = A_2^{1/2} C_3^{1/2}. \tag{86}
$$

As all operators commute, these inequalities, together with $\rho \leq C_1$, imply that $\rho \leq A_1^{1/2} A_2^{1/4} C_2^{1/4}$. Repeating this for the remaining PSD constraints in the dual problem we find that $\rho \leq A_1^{1/2} \ldots A_k^{1/2^k} \sigma^{1/2^k}$ or equivalently $\rho\sigma^{-1/2^k} \leq A_1^{1/2} \ldots A_k^{1/2^k}$. Noting that $-\alpha_k/2^k = 1 - \alpha_k$, by taking both sides of the inequality to the power of α_k we arrive at

$$
\rho^{\alpha_k} \sigma^{1-\alpha_k} \leq A_1^{\alpha_k/2} \ldots A_k^{\alpha_k/2^k}. \tag{87}
$$

It follows that

$$
\begin{aligned}
\mathrm{Tr}\left[\rho^{\alpha_k}\sigma^{1-\alpha_k}\right] &\leq \mathrm{Tr}\left[A_1^{\alpha_k/2} \ldots A_k^{\alpha_k/2^k}\right] \\
&\leq \sum_{i=1}^k \frac{\alpha_k}{2^i} \mathrm{Tr}\left[A_i\right] \\
&= \frac{1}{2^k - 1} \sum_{i=1}^k 2^{k-i} \mathrm{Tr}\left[A_i\right],
\end{aligned} \tag{88}
$$

where the second line follows from the arithmetic-geometric mean inequality. Thus, when $[\rho,\sigma]=0$ we know that $D_{(\alpha_k)}(\rho\|\sigma) \geq \frac{1}{\alpha_k-1}\log \mathrm{Tr}\left[\rho^{\alpha_k}\sigma^{1-\alpha_k}\right]$.

It remains to show that there always exists a feasible point that achieves this bound. For this we choose $A_1 = A_2 = \cdots = A_k = \rho^{\alpha_k}\sigma^{1-\alpha_k}$. It can be verified that this choice satisfies the inequality

$$\rho \leq A_1\#(A_2\#(\ldots\#(A_k\#\sigma)\ldots)) \tag{89}$$

as well as the other constraints of the dual form (77). Therefore, there exists a feasible point of (77) achieving the lower bound $\mathrm{Tr}\left[\rho^{\alpha_k}\sigma^{1-\alpha_k}\right]$ and so the result follows.

Additional Lemmas

Lemma 1. For each $\omega \in [3/4, \frac{2+\sqrt{2}}{4}]$ and each $x\in\{0,1\}$, there exists a state $\rho_{Q_AQ_BE} \in \mathscr{D}(\mathbb{C}^2\otimes\mathbb{C}^2\otimes\mathbb{C}^2)$ and POVMs $\{\{M_{a|x}\}_a\}_x, \{\{N_{b|y}\}_b\}_y$ such that the system $(\rho_{Q_AQ_B}, \{\{M_{a|x}\}_a\}_x, \{\{N_{b|y}\}_b\}_y)$ achieves a CHSH score of ω and

$$\overline{H}_2^{\uparrow}(A|X=x,E) = -\log\left(\frac{1}{2}+\sqrt{4\omega(1-\omega)-\frac{1}{2}}\right), \tag{90}$$

where A denotes the classical register recording the outcomes of the measurements $\{M_{a|x}\}_a$ on the system Q_A.

Proof. To show this we exhibit an explicit set of states and measurements, the choices of which are inspired by [16, 20]. Let $\rho_{Q_AQ_BE} = |\psi\rangle\langle\psi|$ where

$$|\psi\rangle = \sqrt{\frac{\lambda}{2}}(|00\rangle+|11\rangle)\otimes|0\rangle + \sqrt{\frac{1-\lambda}{2}}(|00\rangle-|11\rangle)\otimes|1\rangle \tag{91}$$

for some $\lambda\in[1/2,1]$. Furthermore, let

$$\begin{aligned} M_{0|0} &= \frac{I+\sigma_z}{2} \\ M_{0|1} &= \frac{I+\sigma_x}{2} \\ N_{0|0} &= \frac{I+\cos(\theta)\sigma_z+\sin(\theta)\sigma_x}{2} \\ N_{0|1} &= \frac{I+\cos(\theta)\sigma_z-\sin(\theta)\sigma_x}{2} \end{aligned} \tag{92}$$

with $\theta = \mathrm{atan}(2\lambda-1)$. A direct calculation shows that measuring these POVMs with the reduced state $\rho_{Q_AQ_B}$ results in an expected CHSH score of $\omega = \frac{1}{2}+\sqrt{1-2\lambda(1-\lambda)}$.

Now in [22] it was shown that the optimized Petz conditional entropies had an explicit form

$$\overline{H}_\alpha^{\uparrow}(A|E) = \frac{\alpha}{1-\alpha}\log\mathrm{Tr}\left[\mathrm{Tr}_A\left[\rho_{AE}^{\alpha}\right]^{1/\alpha}\right]. \tag{93}$$

Computing this quantity for $\alpha=2$ and the cq-state ρ_{AE}, where A is the register recording the outcome of the measurement $\{M_{a|0}\}$ on the system Q_A, we find that

$$\overline{H}_2^{\uparrow}(A|X=0,E) = -\log\left(\frac{1}{2}+\sqrt{(1-\lambda)\lambda}\right). \tag{94}$$

Solving the equation $\omega = \frac{1}{2}+\sqrt{1-2\lambda(1-\lambda)}$ for λ and substituting into the above expression we arrive at the expression stated in the lemma. For the case $X=1$ we can repeat the above argument with

measurements defined by the projectors

$$\begin{aligned} M_{0|0} &= \frac{I-\sigma_x}{2} \\ M_{0|1} &= \frac{I+\sigma_z}{2} \\ N_{0|0} &= \frac{I+\cos(\theta)\sigma_z - \sin(\theta)\sigma_x}{2} \\ N_{0|1} &= \frac{I-\cos(\theta)\sigma_z - \sin(\theta)\sigma_x}{2}. \end{aligned} \tag{95}$$

Corollary 1. Let $\Sigma_{CHSH}(\omega)$ be the collection of tuples $(Q_A, Q_B, E, |\psi\rangle\langle\psi|, \{M_{a|x}\}, \{N_{b|y}\})$ such that on expectation the bipartite system $(\mathrm{Tr}_E[|\psi\rangle\langle\psi|], \{M_{a|x}\}, \{N_{b|y}\})$ achieves a CHSH score of ω. Then for each $\omega \in [3/4, \frac{2+\sqrt{2}}{4}]$ and each $x \in \{0,1\}$ we have

$$\inf_{\Sigma_{CHSH}(\omega)} \overline{H}_2^{\uparrow}(A|X=x, E) = -\log\left(\frac{1}{2} + \sqrt{4\omega(1-\omega) - \frac{1}{2}}\right), \tag{96}$$

where A denotes the classical register recording the outcomes of the measurement $\{M_{a|x}\}_a$ on the system Q_A.

Proof. In [19] the authors showed that for each $x \in \{0,1\}$ and each $\omega \in [3/4, \frac{2+\sqrt{2}}{4}]$ we have

$$\inf_{\Sigma_{CHSH}(\omega)} H_{\min}(A|X=x, E) = -\log\left(\frac{1}{2} + \sqrt{4\omega(1-\omega) - \frac{1}{2}}\right). \tag{97}$$

Now as $\overline{H}_2^{\uparrow}(A|X=x,E) \geq H_{\min}(A|X=x,E)$ we must have $\inf_{\Sigma_{CHSH}(\omega)} \overline{H}_2^{\uparrow}(A|X=x,E) \geq -\log(\frac{1}{2} + \sqrt{4\omega(1-\omega) - \frac{1}{2}})$. However, by Lemma 1 we know there exists an explicit strategy achieving this lower bound and so we have equality.

The following lemma provides a useful characterization of positive semidefiniteness for block matrices.

Lemma 2 (Schur complement). Let $A, B, C \in \mathscr{L}(\mathcal{H})$. Then the following are all equivalent:

1. $\begin{pmatrix} A & B \\ B^* & C \end{pmatrix} \geq 0$.
2. $A \geq 0$, $(I - AA^{-1})B = 0$ and $C \geq B^* A^{-1} B$.
3. $C \geq 0$, $(I - CC^{-1})B^* = 0$ and $A \geq BC^{-1}B^*$.

Furthermore, if we restrict to positive-definite matrices then the following are equivalent:

1. $\begin{pmatrix} A & B \\ B^* & C \end{pmatrix} > 0$.
2. $A > 0$ and $C > B^* A^{-1} B$.
3. $C > 0$ and $A > BC^{-1}B^*$.

The following lemma relates block positive semidefinite matrices to the matrix geometric mean.

Lemma 3. Let $A, B \in \mathscr{P}(\mathcal{H})$ and $T \in \mathscr{H}(\mathcal{H})$. Then $A \# B \geq T \iff \exists W \in \mathscr{H}(\mathcal{H})$ such that $W \geq T$ and

$$\begin{pmatrix} A & W \\ W & B \end{pmatrix} \geq 0. \tag{98}$$

Proof. It is well-known that (see e.g., [1, Proposition 3.3.4]) that for $A, B \in \mathscr{P}(\mathcal{H})$ and $W \in \mathscr{H}(\mathcal{H})$ then $\begin{pmatrix} A & W \\ W & B \end{pmatrix} \geq 0 \implies A\#B \geq W$. Therefore if in addition $W \geq T$ we have $A\#B \geq T$. Additionally, they also show that $\begin{pmatrix} A & A\#B \\ A\#B & B \end{pmatrix} \geq 0$ and so the converse holds also.

References

[1] Hiai, F. Matrix analysis: matrix monotone functions, matrix means, and majorization. *Interdisciplinary Information Sciences* **16**, 139–248 (2010).

[2] Petz, D. Quasi-entropies for finite quantum systems. *Reports on Mathematical Physics* **23**, 57–65 (1986).

[3] Müller-Lennert, M., Dupuis, F., Szehr, O., Fehr, S. & Tomamichel, M. On quantum Rényi entropies: A new generalization and some properties. *Journal of Mathematical Physics* **54**, 122203 (2013).

[4] Wilde, M. M., Winter, A. & Yang, D. Strong converse for the classical capacity of entanglement-breaking and hadamard channels via a sandwiched rényi relative entropy. *Communications in Mathematical Physics* **331**, 593–622 (2014).

[5] Umegaki, H. Conditional expectation in an operator algebra, iv (entropy and information). In *Kodai Mathematical Seminar Reports*, vol. 14, 59–85 (Department of Mathematics, Tokyo Institute of Technology, 1962).

[6] Matsumoto, K. A new quantum version of f-divergence. In *Nagoya Winter Workshop: Reality and Measurement in Algebraic Quantum Theory*, 229–273 (Springer, 2015).

[7] Belavkin, V. P. & Staszewski, P. C*-algebraic generalization of relative entropy and entropy. In *Annales de l'IHP Physique theorique*, vol. 37, 51–58 (1982).

[8] Berta, M., Fawzi, O. & Tomamichel, M. On variational expressions for quantum relative entropies. *Letters in Mathematical Physics* **107**, 2239–2265 (2017).

[9] Pironio, S., Navascués, M. & Acín, A. Convergent relaxations of polynomial optimization problems with noncommuting variables. *SIAM Journal on Optimization* **20**, 2157–2180 (2010).

[10] Bunce, J. W. Models for n-tuples of noncommuting operators. *Journal of functional analysis* **57**, 21–30 (1984).

[11] Dupuis, F., Fawzi, O. & Renner, R. Entropy accumulation. *Communications in Mathematical Physics* **379**, 1–47 (2020).

[12] Dupuis, F. & Fawzi, O. Entropy accumulation with improved second-order term. *IEEE Transactions on information theory* **65**, 7596–7612 (2019).

[13] Arnon-Friedman, R., Dupuis, F., Fawzi, O., Renner, R. & Vidick, T. Practical device-independent quantum cryptography via entropy accumulation. *Nature communications* **9**, 459 (2018).

[14] Arnon-Friedman, R., Renner, R. & Vidick, T. Simple and tight device-independent security proofs. *SIAM Journal on Computing* **48**, 181–225 (2019).

[15] Brown, P. J., Ragy, S. & Colbeck, R. A framework for quantum-secure device-independent randomness expansion. *IEEE Transactions on Information Theory* **66**, 2964–2987 (2019).

[16] Pironio, S. *et al.* Device-independent quantum key distribution secure against collective attacks. *New Journal of Physics* **11**, 045021 (2009).

[17] Tan, E. Y.-Z., Schwonnek, R., Goh, K. T., Primaatmaja, I. W. & Lim, C. C.-W. Computing secure key rates for quantum key distribution with untrusted devices. *Preprint at arXiv:1908.11372* (2019).

[18] Assad, S. M., Thearle, O. & Lam, P. K. Maximizing device-independent randomness from a Bell experiment by optimizing the measurement settings. *Physical Review A* **94**, 012304 (2016).

[19] Masanes, L., Pironio, S. & Acín, A. Secure device-independent quantum key distribution with causally independent measurement devices. *Nature Communications* **2**, 238 (2011).

[20] Murta, G., van Dam, S. B., Ribeiro, J., Hanson, R. & Wehner, S. Towards a realization of device-independent quantum key distribution. *Quantum Science and Technology* **4** (2019).

[21] Tomamichel, M. *Quantum Information Processing with Finite Resources: Mathematical Foundations*, vol. 5 (Springer, 2015).

[22] Tomamichel, M., Berta, M. & Hayashi, M. Relating different quantum generalizations of the conditional rényi entropy. *Journal of Mathematical Physics* **55**, 082206 (2014).
